# Insights into estuary habitat loss in the western United States using a new method for mapping maximum extent of tidal wetlands

**DOI:** 10.1371/journal.pone.0218558

**Published:** 2019-08-14

**Authors:** Laura S. Brophy, Correigh M. Greene, Van C. Hare, Brett Holycross, Andy Lanier, Walter N. Heady, Kevin O’Connor, Hiroo Imaki, Tanya Haddad, Randy Dana

**Affiliations:** 1 Institute for Applied Ecology, Corvallis, Oregon, United States of America; 2 NOAA Fisheries, Northwest Fisheries Science Center, Seattle, Washington, United States of America; 3 Pacific States Marine Fisheries Commission, Portland, Oregon, United States of America; 4 Oregon Dept. of Land Conservation and Development, Salem, Oregon, United States of America; 5 The Nature Conservancy, Santa Cruz, California, United States of America; 6 Moss Landing Marine Labs, Moss Landing, California, United States of America; 7 Pacific Spatial Solutions Inc., Seattle, Washington, United States of America; Universidade de Aveiro, PORTUGAL

## Abstract

Effective conservation and restoration of estuarine wetlands require accurate maps of their historical and current extent, as well as estimated losses of these valued habitats. Existing coast-wide tidal wetland mapping does not explicitly map historical tidal wetlands that are now disconnected from the tides, which represent restoration opportunities; nor does it use water level models or high-resolution elevation data (e.g. lidar) to accurately identify current tidal wetlands. To better inform estuarine conservation and restoration, we generated new maps of current and historical tidal wetlands for the entire contiguous U.S. West Coast (Washington, Oregon, and California). The new maps are based on an Elevation-Based Estuary Extent Model (EBEEM) that combines lidar digital elevation models (DEMs) and water level models to establish the maximum historical extent of tidal wetlands, representing a major step forward in mapping accuracy for restoration planning and analysis of wetland loss. Building from this new base, we also developed an indirect method for mapping tidal wetland losses, and created maps of these losses for 55 estuaries on the West Coast (representing about 97% of historical West Coast vegetated tidal wetland area). Based on these new maps, we estimated that total historical estuary area for the West Coast is approximately 735,000 hectares (including vegetated and nonvegetated areas), and that about 85% of vegetated tidal wetlands have been lost from West Coast estuaries. Losses were highest for major river deltas. The new maps will help interested groups improve action plans for estuarine wetland habitat restoration and conservation, and will also provide a better baseline for understanding and predicting future changes with projected sea level rise.

## Introduction

Estuaries have long attracted human settlement due to their proximity to the ocean and their abundance of productive flat land, and human modifications to the landscape have resulted in extensive loss of estuarine wetlands [1]. But what has been the actual extent of tidal wetland loss? The answer to this important question is obscured by centuries-long change, lack of data on historical estuary extents, and absence of records on wetland conversion. Nevertheless, estimates of the historical maximum extent of tidal wetlands are important for many reasons. These data provide information for assessing loss of function of tidal wetlands, including ecosystem services [2] such as flood mitigation [3], carbon storage [4, 5] and contaminant sequestration [6]. Because tidal wetlands provide nursery rearing habitat critical to maintaining populations of marine fishes and invertebrates [2, 7, 8], historical estuary extents provide a baseline for understanding habitat changes that impact estuarine dependent species. Information on historical wetland extents helps facilitate identification of sites that are geomorphically compatible with habitat restoration. Finally, historical data provides a baseline to determine risk to tidal wetlands and their ecosystem services with projected future sea level rise [9].

Some efforts have been made to estimate tidal wetland losses across large spatial extents (many estuaries). For example, Carle [10] used national land cover data to infer wetland loss from 1998–2004 across North Carolina. Stedman and Dahl [11] inferred recent estuary loss (1998–2004) from changes in remotely sampled sites using USFWS wetlands status and trends monitoring program. Gosselink and Baumann [12] estimated loss by comparing USDA survey maps dating as far back as 1922 with recent surveys using different methods. While all these estimates can provide sound estimates of recent estuarine wetland loss, they nevertheless miss extensive wetland loss resulting from the earlier stages of estuary conversion, which in the United States started in the 1600s for the Atlantic Coast and the 1800s for the Pacific Coast. Such omissions illustrate the problem of "shifting baselines" [13] and obscure the true magnitude of change.

On the Pacific Coast, several efforts have focused on determining historical estuarine footprints for smaller spatial extents [14–17]. These efforts have often taken advantage of T-sheets produced in the 19^th^ century as part of the United States Coast Survey. However, due to subjectivity of individual surveyors, resulting T-sheets vary in the level of detail. This, combined with the absence of a standardized legend, can make T-sheet interpretation difficult [18]. Furthermore, mapping efforts from T-sheets are by nature piecemeal (single estuary systems mapped), have employed slightly different methods to convert T-sheets to historical maps, and rely on datums that are not necessarily available for estuaries outside the range of the initial survey. Hence, there are no consistent maps of historical estuary wetlands across large areas, hindering our ability to examine the impacts of wetland loss and appropriate management actions at these extents.

We take a new approach to mapping estuary extent and wetland loss by logically following through with a simple observation: tidal wetlands are defined by the repeated action of tidal inundation upon the wetland surface. Accordingly, the maximum extent of tidal wetlands can be determined using the combination of land surface elevation data and analyses of tidal inundation frequency. In this paper, we lay out a method of characterizing the maximum extent of tidal wetlands for Washington, Oregon, and California. This method (the “Elevation-Based Estuary Extent Model” or EBEEM), combines ground-truthed evaluations, analyses of lidar-derived Digital Elevation Models (DEMs) and tide gauge data for the tri-state coast, and integration of other mapping products to determine the historical footprint of estuaries. To validate our approach, we compare our results with other system-specific efforts in large estuaries. We then combine this geomorphically-based estimate of historical estuary extent with data on current tidal wetlands from the National Wetlands Inventory to infer tidal wetland loss.

## Materials and methods

### Study area

The study area comprises all estuaries on the Pacific Coast of the contiguous United States (Washington, Oregon, and California). The spatial domain includes portions of the fjord complex of the Salish Sea, the immense Columbia River estuary, and the extensive Sacramento/San Joaquin River delta connected with San Francisco Bay. We define estuaries generally following Pritchard [19] and Wolanski [20] as a partially enclosed body of water or wetland that periodically receives freshwater and seawater inputs and extends from its connection to the ocean to the limit of tidal influence, defined by salinity gradients or tidal inundation. This definition therefore includes fjords, large riverine deltas, coastal creek mouths, bar-built and lagoonal estuaries, and large embayments, as well as systems that span the entire range of salinity from tidal freshwater (0 PSU) to hypersaline (>40 PSU). Examples of all these features exist across the United States Pacific Coast.

### Data sources

**Fig 1:**
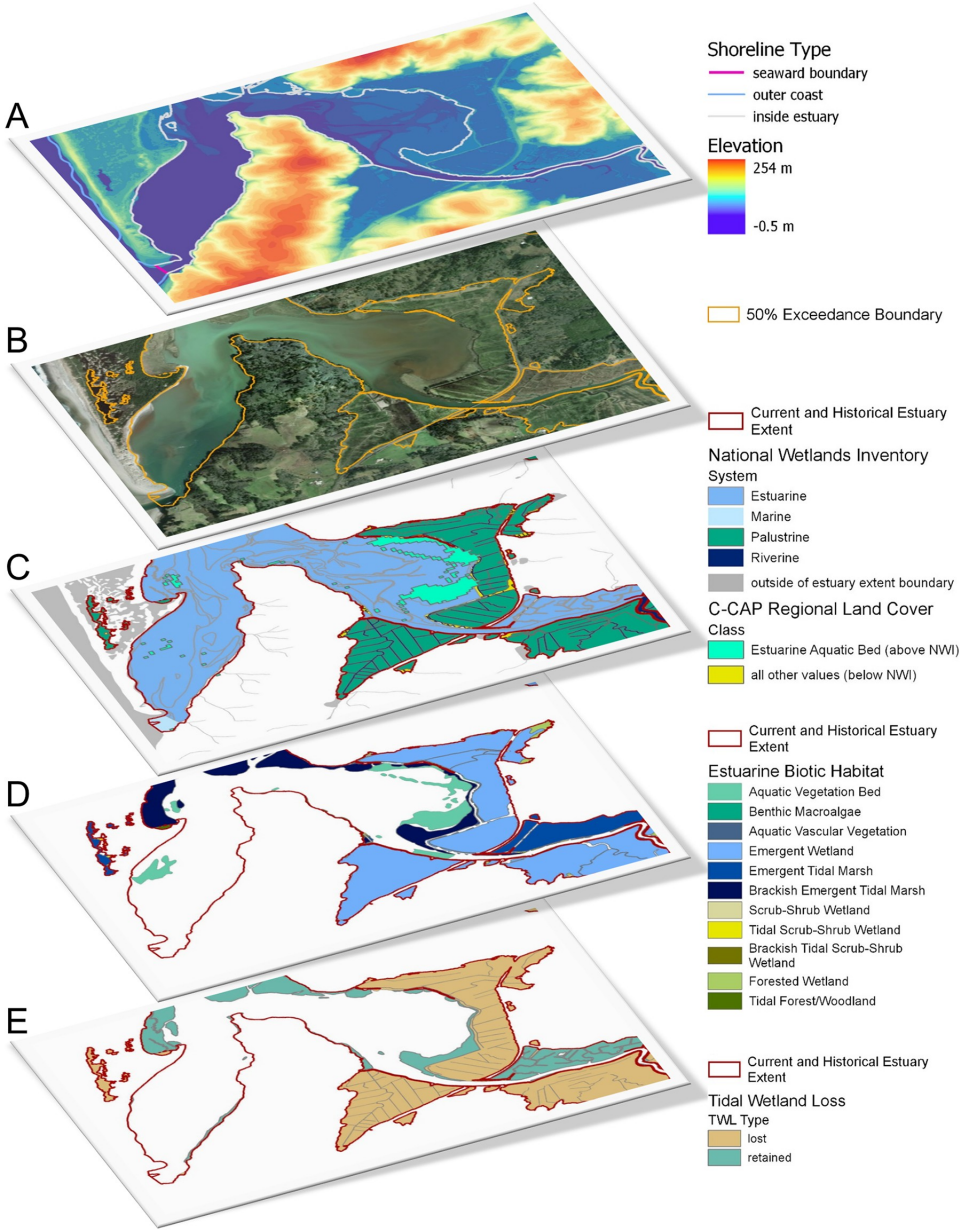
Schematic of data sources used in EBEEM analysis and outlined in Table 1. A) Elevation (topography and bathymetry) from lidar DEM and modified CUSP shoreline data. B) 50% exceedance elevation boundary derived from DEM surface. C) National Wetlands Inventory (NWI) and C-CAP Regional Land Cover data within estuary extent layer. D) West Coast USA Estuarine Biotic Habitat layer derived from NWI and C-CAP. E) Indirect Assessment of West Coast USA Tidal Wetland Loss layer.

In order to produce maps of historical estuary extent and wetland loss, we incorporated a number of datasets (Table 1). These were used to produce maps of elevation, estuarine shoreline, and extent of estuarine wetlands (Table 1 and Fig 1), and uses of these inputs are described further below.

**Table 1:** Data sources for estimation of elevation-based estuary extent model (EBEEM) and wetland loss, and how data were used in GIS modeling. See S1 File for URLs to elevation data sources.

Data Category	Dataset	Source	Resolution or Scale	Publication Year	Input Into
Elevation	NOAA Coastal DEM	National Geophysical Data Center (NOAA)	~10 m	2008–2012	minus 4m MLLW^1^^,^^2^
	Coastal Inundation DEM	Office For Coastal Management (NOAA)	~5 m	2007–2013	50% exceedance^1^
	Clallam County (2001), Olympic Peninsula (2005), Hoh River Watershed (2012–2013)	Puget Sound LiDAR Consortium	~0.9–1.8 m (3–6 feet)	2001–2013	50% exceedance^1^
	Oregon Coast LiDAR (North & South)	Oregon Department of Geology and Mineral Industries (DOGAMI)	~0.27 m	2008–2009	50% exceedance^2^
	Puget Sound Combined Bathymetry and Topography	University of Washington	10 m	2000, 2005	50% exceedance, minus 4m MLLW^1^
	A Continuous Surface Elevation Map for Modeling	California Department of Water Resources, Bay-Delta Office	2–10 m	2012	50% exceedance^1^
	2009–2011 CA Coastal TopoBathy Merged DEM	California Coastal Conservancy	10 m	2013	50% exceedance^1^
	Suisun Bay and Delta Bathymetry (USGS)	United States Geological Survey	10 m	2003	50% exceedance^1^
Shoreline	Continually Updated Shoreline Product (CUSP)	NOAA National Geodetic Survey	1:1,000–1:24,000	2016	shoreline^1^
	Oregon Continually Updated Shoreline Product	Oregon Coastal Management Program	1:1,000–1:24,000	2015	shoreline^1^
Extent/ Habitat	National Wetlands Inventory	U.S. Fish and Wildlife Service	1:24,000	2016	estuary extent and estuarine biotic habitat^1^^,^^2^
	C-CAP Land Cover Atlas	NOAA Coastal Services Center	1:100,000	2010, 2011	estuarine biotic habitat^1^^,^^2^
	An Inventory and Classification of U.S. West Coast Estuaries	The Nature Conservancy	N/A	2015	estuary extent (CA lagoons only)^1^
	USACOE 50% Annual Exceedance Probability Stage for Survival Benefit Unit for the Lower Columbia River Estuary	PC Trask & Associates	1 m	2013	estuary extent (Columbia River only)^1^

^1^ Pacific Marine and Estuarine Fish Habitat Partnership

^2^ Oregon Coastal Management Program

### Mapping the upslope elevation for tidal wetlands

The first step in mapping the current and historical extent of tidal wetlands was to determine an appropriate elevational boundary for their upslope or landward extent. Landward boundaries of estuaries and tidal wetlands are the subject of some debate, and have been based on the upper limit of salinity signals [21] ecological and physical processes [22] and elements of marine hydrography such as the limits of tidal inundation [20, 23, 24]. However, in regulatory and wetland functional assessment settings, the upslope extent of tidal wetlands is commonly defined as the elevation of the highest tides of the year (e.g. “annual high tide” or “king tide”) [24–26]. We therefore used the same definition in our mapping effort.

#### Determining annual high tide elevations

**Fig 2:**
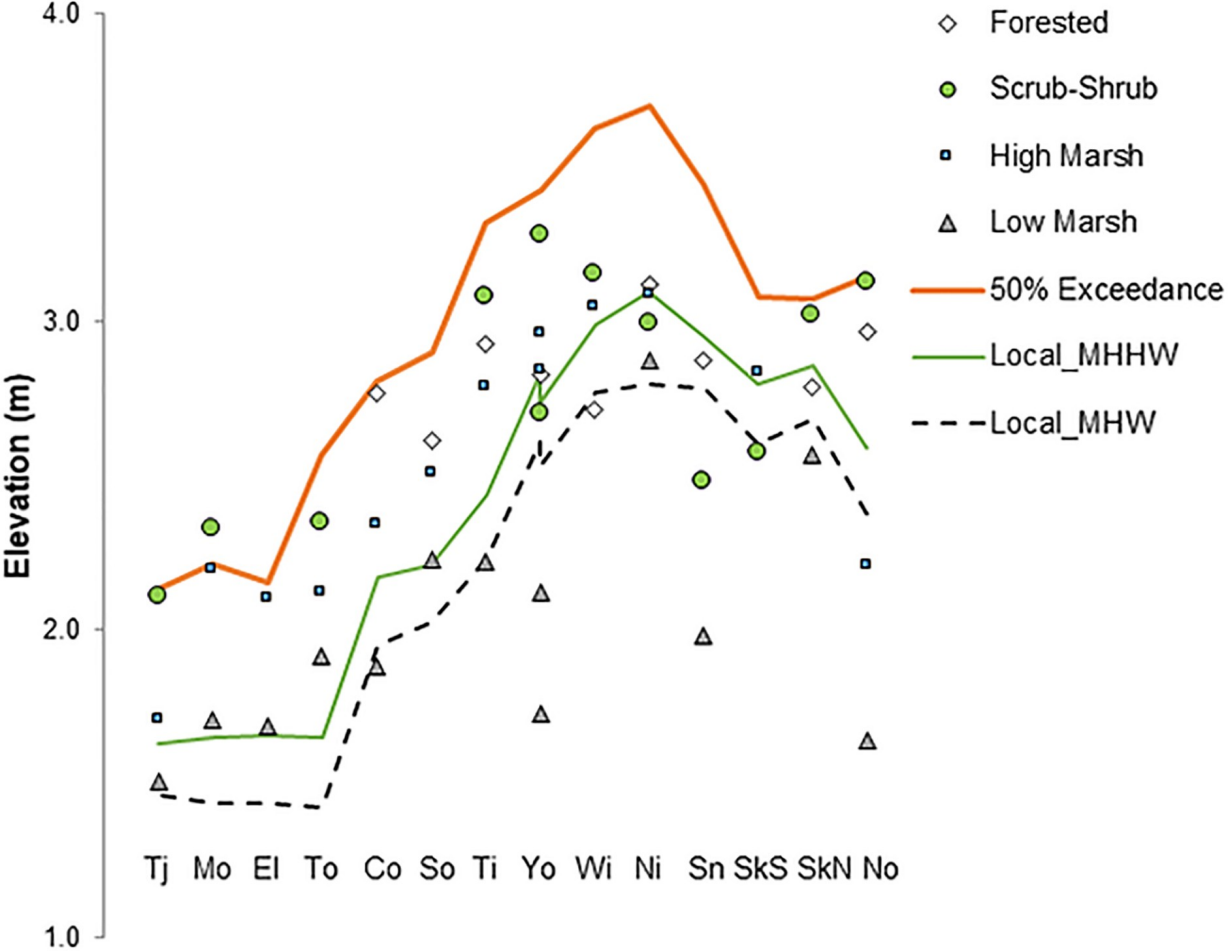
Observations of elevational data (relative to NAVD88) from 14 estuary systems across California, Oregon, and Washington. Estuaries (south to north in order from left to right) evaluated include Tijuana (TJ), Morro Bay (Mo), Elkhorn Slough (El), Tomales Bay (To), Coquille (Co), South Slough (So), Tillamook (Ti), Young’s Bay (Yo), Willapa Bay (Wi), Nisqually (Ni), Snohomish (Sn), South Fork Skagit (SkS), North Fork Skagit (SkN), and Nooksack (No). In contrast to MHHW (green line) and MHW (black line), 50% exceedance elevation (orange line) consistently surpasses average lidar-based elevations of all four tidal wetland types (other symbols) which were verified as tidally inundated in the field (S2 File).

Our goal was to determine a broadly applicable tidal datum that would represent the annual high tide elevation, and that could thereby be used to map the upslope extent for a range of estuary types and geographies. However, previous definitions of the maximum extent of estuarine influence are problematic. The Coastal and Marine Ecological Classification System (CMECS), the current U.S. Federal Geographic Data Committee’s current standard for estuary habitat classification [27], proposes mean higher high water (MHHW) as the upslope boundary for the Estuarine Coastal Intertidal tidal zone (>0.5 PSU). However, major tidal wetland classes of the West Coast occur above MHHW [28–31] (Fig 2)—and as a result, the MHHW boundary conflicts with the boundaries used in ecological, regulatory and wetland assessment contexts. Although CMECS’ Estuarine Coastal subsystem also includes a “supratidal” zone above MHHW, its definition (the zone of “wave splash and overwash”) [27] is not appropriate for intertidal wetlands of the West Coast. For the Estuarine Tidal Riverine Coastal subsystem (<0.5 PSU, i.e. freshwater tidal wetlands), CMECS relies upon “extreme high water of spring tides” (EHWS) as the upslope boundary [27]. However, this subsystem is not applicable to West Coast estuarine wetlands with brackish salinity regimes (which are prevalent in most estuaries); moreover, the National Oceanic and Atmospheric Administration (NOAA) does not provide elevations for EHWS at West Coast tide stations. Published datums for NOAA’s West Coast tidal stations generally include only three high water datums: MHHW, highest observed tide (HOT), and highest astronomical tide (HAT). Since HOT and HAT report the highest observations across an entire 19-year National Tidal Datum Epoch, they may be higher than annual high tide levels. In addition, as individual extreme water level observations across a 19-year epoch, they are less appropriate as ecological boundaries compared to tidal datums expressing the probability of inundation.

To address these problems, we selected NOAA’s Extreme Water Levels (EWL) analysis (http://tidesandcurrents.noaa.gov/est/, [32]), which provides several exceedance level water elevations (1%, 10%, 50%, and 99%). These represent annual probabilities of water levels exceeding the given elevation–probabilities of 1%, 5%, 50%, and 99% respectively. For example, the 50% exceedance elevation is 1.99 m above Mean Sea Level, which is 0.81 m above Mean Higher High Water (MHHW) at South Beach, Oregon for the 1983–2001 tidal epoch. This means it is probable a tide will exceed 0.81 m above MHHW in half of all years, or about once every two years. Further, the 50% exceedance is 0.44 m below HOT (although 0.12 m above HAT). We used 50% exceedance elevation in conjunction with high-resolution elevation data from lidar DEMs to map the maximum annual extent of tidal inundation across the landscape.

#### Validating the upslope estuary boundary

Based on the definition of tidal wetlands above (wetlands inundated by the highest annual tide), we tested the 99% and 50% exceedance elevations for possible use in our mapping. Initial analysis of Oregon estuaries [33] indicated that the 99% exceedance elevation was too low and thus inundated too frequently, while the 50% exceedance elevation might be an appropriate tidal wetland boundary. We analyzed the 50% exceedance elevation as a potential upslope boundary at sites within 14 estuaries spanning the Pacific Coast from southern California to Northern Washington. At these sites, we obtained field observations of inundation, wetland type, year-round local water level data from electronic dataloggers, and ground surface elevation from the lidar DEM (S2 File). We determined that the 50% exceedance contour was a good fit for the approximate maximum extent of tidal wetlands, particularly in contrast to the MHHW datum (Fig 2). Elevations of forested, scrub-shrub, and high marsh tidal wetlands tended to occur between MHHW and 50% exceedance boundaries throughout the latitudinal range of these validation sites. Although ground surfaces at the 50% exceedance elevation might theoretically be inundated less than annually, our field data (Fig 2) and the validation procedures above indicated these areas actually inundated at least annually and often more than once a year. Therefore, we selected and mapped the 50% exceedance contour as the upper boundary for tidal wetlands.

#### Determining land-surface elevations

The EBEEM method required data on land surface elevations (topography) to compare with tidal elevations. To create the land surface, we combined lidar elevation data from multiple sources (see Table 1). Using GIS, we incorporated multiple lidar datasets (vertical datum NAVD 88) into a coast-wide merged bare earth DEM.

#### Adjusting vertical datums within estuaries

**Fig 3:**
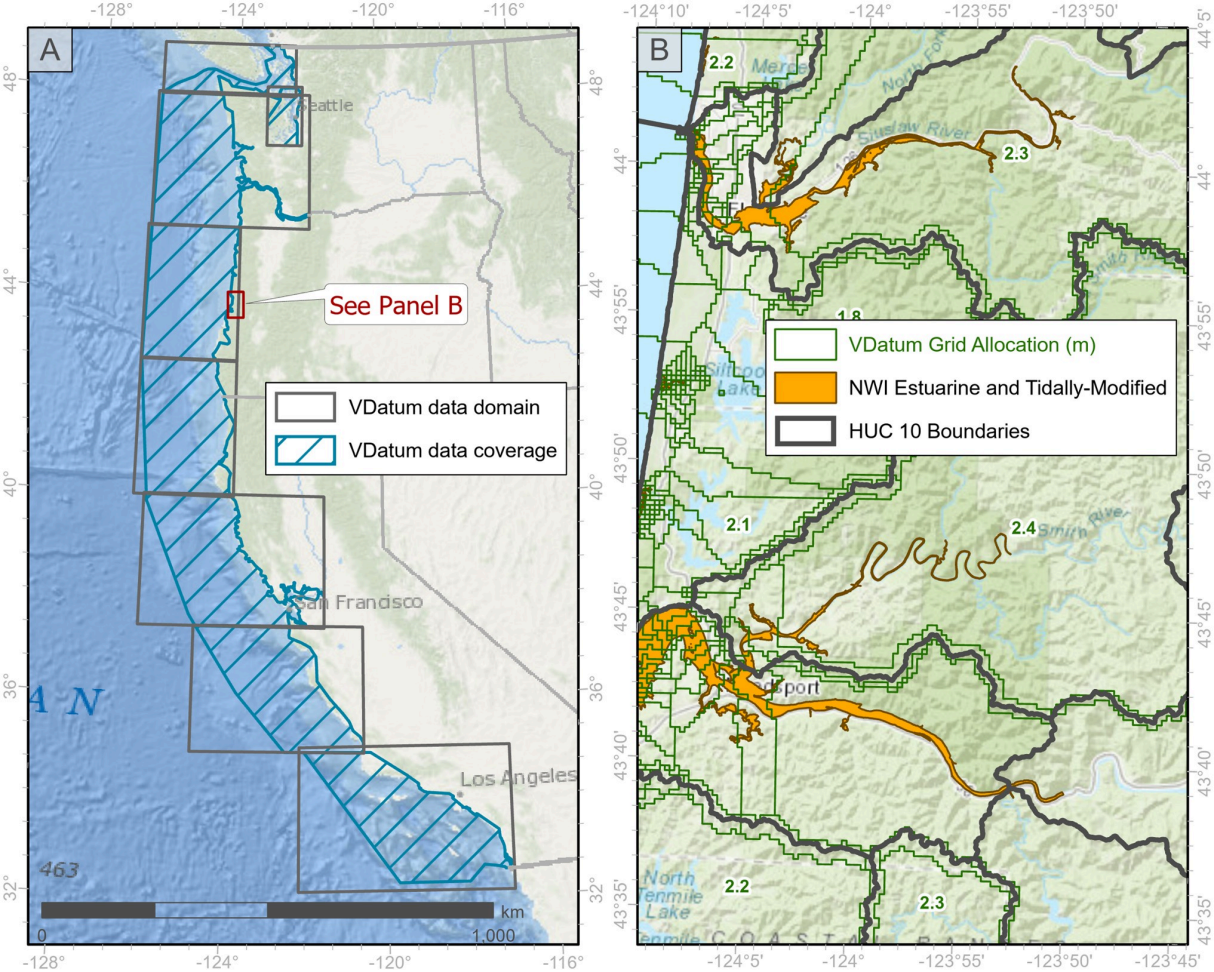
Extent of VDatum coverage for the Pacific Coast and its estuaries. A) Coverage of VDatum across Washington, Oregon, and California. B) illustration of estuarine areas (in orange) that required spatial interpolation because they lie landward of the range of VDatum data coverage.

The elevation-based modeling approach also required conversions between vertical reference systems (tidal datums for tide gauges and NAVD 88 for the lidar DEMs). We used NOAA’s VDatum tool (http://vdatum.noaa.gov/) to convert between these vertical reference systems. We generated a MHHW raster for the entire coast using VDatum and converted it to the NAVD 88 datum to match the lidar data. VDatum does not fully cover all potentially tidally inundated areas (Fig 3), particularly in coastal river systems. Therefore, we extrapolated VDatum values upriver. We explored several GIS-based extrapolation techniques (including kriging, spline interpolation, trend analysis, and Euclidean allocation), and selected the Euclidean allocation algorithm in ArcGIS for the extrapolation. This method has been previously used in NOAA’s Coastal Inundation Mapping (http://coast.noaa.gov/digitalcoast/training/inundationmap).

We refined this technique by constraining Euclidean Allocations to follow coastal topography. Because the Euclidean allocation algorithm is non-directional, interpolated values sometime overshot watershed boundaries. To prevent this kind of “contamination,” we used HUC 12 and 10 watershed boundaries to “seal” interpolated values. We used HUC 12 polygons to process watersheds where VDatum values were available partially or entirely. For those areas not covered by VDatum, we used HUC10 polygons to extend the analysis extent using interpolated values from the previous step as source values for the Euclidean allocation process.

#### Interpolating 50% exceedance elevations

We generated a vertical height raster that revealed elevation differences between 50% exceedance elevation and MHHW. The 50% exceedance value was calculated using the 22 West Coast tide gauges for which NOAA provides EWL data (“EWL gauges”), and values were interpolated for the estuaries without EWL gauges. For areas between the EWL gauges, the exceedance value was calculated from the relative distance of the intermediate point to the nearest EWL gauges. We interpolated values with a simple spline function [34] (“Spline with Barriers” geoprocessing tool in ArcGIS). This function generates an interpolated surface, and unlike other methods (e.g., Kriging, Natural Neighbor, and spline with/without tension) considers impenetrable barriers such as a landmass fundamental to elevation determinations in estuaries (Fig 3).

We added elevation differences between 50% exceedance level and MHHW to the NAVD 88 translated MHHW raster. Since almost all lidar and DEM products use NAVD 88 as their vertical datum, we used NAVD 88 as a standard vertical datum. We then subtracted the 50% exceedance elevation raster from the NAVD 88 elevation data set. Areas with raster values less than 0 (i.e., lower than the 50% exceedance elevation) were interpreted as historically or currently subject to tidal inundation. We extracted areas that had negative raster values and generated polygons to show areas lower than the 50% exceedance water level.

#### Refining the estuary extent

We amended the base 50% exceedance map based on several additional datasets. The final product was the “West Coast USA Current and Historical Estuary Extent” (hereafter referred to as the “Estuary Extent” or “EBEEM mapping”). In summary, this product is composed of the mapping we generated for areas below the 50% exceedance contour, with additional areas derived from three sources described below: tidal wetlands from the National Wetlands Inventory that fell above (outside) the 50% exceedance contour, additional wetlands within lagoonal estuaries of California, and locally-generated mapping for the Columbia River Estuary based on locally-modeled Columbia River 50% exceedance values.

#### Adding National Wetlands Inventory tidal wetlands above the 50% exceedance contour

Tidal wetlands from National Wetlands Inventory (NWI) that extended beyond the 50% exceedance contour were appended to the dataset. NWI classes appended to the dataset include all Estuarine system classes, as well as any Palustrine and Riverine system classes with tidal modifiers (‘S’, ‘Q’, ‘R’, ‘T’, and ‘V’). We did this because in our experience, it can be safely assumed that the NWI’s tidal classification is conservative, i.e. there is little risk of NWI incorrectly classifying nontidal wetlands as tidal. Also, spatial data analysis during this project showed that there were very few NWI tidal wetlands outside the 50% exceedance contour.

#### Maximum extent in California lagoonal estuaries

Boundaries of lagoonal estuaries along the outer coast of California were delineated using a two-part approach. First, an ArcGIS file was obtained from the Southern California Coastal Water Research Project (SCCWRP) which contained estuarine wetland boundaries for 190 coastal confluences for the southern California bight. The estuary polygons were generated from NWI data based on NWI codes that provided evidence of periodic tidal inundation (e.g., marine and estuarine codes and lacustrine and palustrine codes with tidal regimes). Second, we used aerial imagery to identify and geo-locate a comprehensive inventory of the remaining California estuaries. Again, we used NWI data to determine the extent of the estuary; if NWI was not available or was obviously incorrect, a new polygon was drawn using the current and historical aerial imagery provided by Google Earth Pro and the California Coastal Records Project (http://www.californiacoastline.org/). The maximum extent of each estuary was drawn at the estimated maximum inundation elevation throughout the associated marsh plain when the beach berm was closed. High-water aerial images, wrack lines and changes in vegetation from marsh plain to woody riparian or terrestrial were all utilized as indicators of maximum inundation.

#### Columbia River

The Columbia River estuary has a large river surface elevation gradient, with the 50% exceedance elevation rising from 3.5 m to 8.7 m NAVD 88 along the 235 km of the river’s tidal mainstem from the ocean to Bonneville Dam. This gradient is reflected in the elevations of tidal wetlands, which are extensive across all tidal reaches. Therefore, we did not expect the Columbia River’s single NOAA tide gauge with EWL data (located near the river mouth at Astoria) to provide adequate data for our mapping effort. Instead, we sought locally-developed data for this estuary. Such data were provided to us as a map of the area below the 50% exceedance contour for the entire Columbia River estuary [35]. This map was generated from the U.S. Army Corps of Engineers’ (USACE) models of Columbia River water levels as cited by the Expert Regional Technical Group [36]. We then refined our mapping to include any areas below the 50% exceedance elevation, but beyond the extent of the locally mapped data [35].

### Determining seaward extent

Defining the Estuary Extent required a seaward boundary in addition to a landward elevation contour. The methods used to establish a seaward boundary for estuaries differed by region and estuary type. For estuaries outside of the Salish Sea region, we used a modified version of NOAA’s Continually Updated Shoreline Product (CUSP) and Oregon’s CUSP to establish seaward boundaries at the mouths of the estuaries. If the CUSP shoreline data continued into the estuary, the shoreline feature was split on each side at a point roughly halfway between the narrowest part of the mouth and an imaginary line extension of the outer coast. A straight line was created connecting these two points, and this line was used to extend or trim the Estuary Extent, depending on relative location of the input 50% exceedance feature. If the CUSP shoreline did not extend into the estuary, it served as the seaward extent. For estuaries with jetties, the seaward extent was created by digitizing a straight line between the ends of the jetties.

For non-lagoonal Salish Sea estuaries, the seaward boundary was established at a bathymetric depth contour depicting 4 m below Mean Lower Low Water (MLLW) using the NOAA National Geophysical Data Center’s Integrated Models of Coastal Relief digital elevation models. The 4 m depth contour was selected because it represents the seaward boundary of the Estuarine Coastal subsystem within CMECS [27]. For lagoonal estuaries in the Salish Sea region, the CUSP shoreline was utilized as the seaward boundary.

### Classifying wetlands

We used CMECS [27] to define wetland types within the Estuary Extent described above. A number of classification schemes for estuarine environments exist [23, 37–40]. We used CMECS because it is accepted as a federal standard [27], and because it is hierarchical and provides powerful tools for delineating geomorphic and biotic attributes. These classifications (Table 2) were used to 1) develop the appropriate biogeographic context (based on marine ecoregions) for comparing among estuary systems, 2) assign estuaries into major physiographic classes (embayments, lagoons, major river deltas, and riverine estuaries) of similar geomorphology, and 3) determine biotic subgroups that define ecologically important wetland types within each estuary. As noted in Table 2, Aquatic Setting, Water Column, and Substrate Components of CMECS were not classified in this effort. However, within CMECS’ Aquatic Setting, the Estuary Extent described above maps the extent of the Estuarine System. (Lacustrine and Marine Systems were not mapped, as they are outside the estuary).

**Table 2:** CMECS classifications used in unit definition, and sources of information used to make classifications. Abbreviations: MEOW = Marine Ecoregions of the World, PMEP = Pacific Marine and Estuarine Fish Habitat Partnership, NWI = National Wetlands Inventory, C-CAP = Coastal Change Analysis Program.

CMECS Setting/Component	Unit	Source	Unit Names Present in PMEP Estuary Data
**Biogeographic Setting**			
	Realm	MEOW	Temperate Northern Pacific
	Province	MEOW	Cold Temperate Northeast Pacific, Warm Temperate Northeast Pacific
	Ecoregion	Modified MEOW	Salish Sea, Washington, Oregon, Northern California Coast, Central California, Southern California Bight
**Aquatic Setting**			
	System	NOAA water level models + LIDAR DEM	Estuarine (full historical extent of Estuarine System, including diked/disconnected areas)
**Water Column Component**			
Geoform Component			
	Physiographic Setting	various, PMEP	Embayment/Bay, Lagoonal Estuary, Major River Delta, Riverine Estuary
**Substrate Component**			
Biotic Component			
	Biotic Setting		Benthic/Attached Biota
	Biotic Class	NWI & C-CAP	Aquatic Vegetation Bed, Emergent Wetland, Forested Wetland, Scrub-Shrub Wetland
	Biotic Subclass	NWI	Aquatic Vascular Vegetation, Benthic Macroalgae, Emergent Tidal Marsh, Tidal Forest/Woodland, Tidal Scrub-Shrub Wetland
	Biotic Group	NWI	Brackish Emergent Tidal Marsh, Brackish Tidal Forest/Woodland, Brackish Tidal Scrub-Shrub Wetland

### Determining wetland loss

We used an indirect method to estimate tidal wetland losses since European settlement for 55 major estuaries of the West Coast, comprising 97% of West Coast historical tidal wetland area. Loss was assessed for emergent, shrub, and forested tidal wetlands, since these classes are most commonly prioritized for restoration. Losses were determined for these tidal wetland classes as a whole, and not broken down by wetland class, because determining loss by wetland class requires additional data on historic vegetation that are not currently available coastwide.

Wetland loss was defined as those areas that 1) were tidal wetlands prior to European settlement, but 2) are no longer tidal wetlands. Condition (1) was inferred as areas below the 50% exceedance contour, and condition (2) was estimated as those areas below the 50% exceedance contour that are classified as nontidal wetlands in NWI or absent from NWI entirely (and therefore upland). Our analysis method is therefore indirect; rather than directly identifying areas that have been lost, the method compares the full historical extent of tidal wetlands (the EBEEM mapping described above) to existing mapping of current tidal wetlands from the NWI. The basic geoprocessing approach (S3 File) was to intersect the Estuary Extent layer with the NWI. Areas that were classified as tidal wetlands in NWI (either in the estuarine system in NWI, or in other systems with tidal modifiers) were considered “retained.” Other areas–classified in NWI as either upland or nontidal wetlands–were considered “lost” (Table 3).

**Table 3:** Summary of tidal wetland loss classification for National Wetlands Inventory (NWI) wetland types. Blank cells represent attribute combinations not present in the database. "Lost" indicates wetlands that were probably once vegetated tidal wetlands, but are no longer in that category. "Retained" indicates areas that were probably historically vegetated tidal wetlands and still remain in that category. "NA" indicates wetlands omitted from the analysis, because the analysis is limited to emergent (EM), scrub-shrub (SS) and forested (FO) tidal wetland vegetation classes.

	Vegetated (EM, SS, or FO)				Non-vegetated or Aquatic Bed			
	Nontidal water regime		Tidal water regime		Nontidal water regime		Tidal water regime	
NWI System	Diked/ drained/ farmed	Not diked/ drained/ farmed	Diked/ drained/ farmed	Not diked/ drained/ farmed	Diked/ drained/ farmed	Not diked/ drained/ farmed	Diked/ drained/ farmed	Not diked/ drained/ farmed
Marine								NA
Riverine				retained				NA
Estuarine			lost	retained			lost	NA
Palustrine	lost	lost	lost	retained	lost	lost	lost	NA
Lacustrine	lost	lost	lost	retained	lost	lost	lost	NA
None (uplands)	All are considered "lost" except as described in project metadata							

This assessment’s methods are applicable only to vegetated tidal wetlands in the emergent, scrub-shrub and forested classes, and not to aquatic beds, mud flats, open water areas, or other wetland types. These latter wetland types are not included in the analysis. We also excluded all lagoonal estuaries from the analysis. In lagoonal estuaries, maximum water levels may occur at times other than high tides, when river flow inundates areas bounded by seasonally forming berms [41]. Hence, in lagoonal estuaries, wetland classes mapped in NWI as non-tidal wetlands could not be used to determine extent of tidal wetland losses. The assessment was limited to larger estuaries (>100 ha historical estuary area), because the scale and methods for the NWI mapping were inadequate for assessment of smaller estuaries. These limitations are discussed further in “Challenges and uncertainties” below.

We compared our results to independent assessments of tidal wetland connection status in three areas: the Lower Columbia River estuary, Oregon’s outer coastal estuaries, and San Francisco Bay. These areas were chosen because they have been well-studied and have high-quality spatial data on tidal connectivity. If our methods are reasonable and appropriate, we would expect the areas classified as “lost” in our assessment to match fairly well with areas mapped as “diked” or “disconnected” in these three datasets.

#### Columbia River Estuary

We compared our results to a spatial dataset called "Extent of Tidal Influence and Tidally Restricted Areas in the Lower Columbia River Estuary (Final Reclassified Data, All Zones)" obtained from the Lower Columbia Estuary Partnership [42]. For comparison to our analysis, areas classified in the LCEP dataset as "significantly restricted tidal" or "completely blocked" were considered "lost"; areas classified in LCEP as "unrestricted tidal" or "partially restricted tidal" were considered "retained." The "partially restricted tidal" areas were described in the LCEP metadata as "areas where culverts, bridges or levee breaches provide fairly unrestricted flow."

#### Oregon outer coast estuaries

We compared our results to the Oregon Coastal Management Program’s (OCMP’s) CMECS datasets (http://www.coastalatlas.net/cmecs) for all 12 Oregon estuaries represented in the OCMP datasets (Nehalem River, Tillamook Bay, Netarts Bay, Nestucca Bay, Salmon River, Siletz Bay, Yaquina Bay, Alsea Bay, Siuslaw River, Umpqua River, Coos Bay, and Coquille River). The OCMP CMECS Aquatic Setting and Biotic Component layers both use an Anthropogenic Impact Modifier (AI07) to designate diked areas. Since the Aquatic Setting and Biotic Component layers differ slightly in extent, these two layers were merged for the analysis. Areas with the AI07 modifier were considered "lost" for comparison with our assessment results; areas without the modifier were considered "retained."

#### San Francisco Bay

We compared our results to the San Francisco Estuary Institute’s Historical and Modern Baylands dataset, SFEI EcoAtlas Version 3.0 [43]. Areas classified in the SFEI dataset as "Diked", "Filled Bayland", "Hillslope", "Muted Tidal Bayland" and "Undefined" were considered "lost"; and “Alluvial Plain”, “Deep Bay”, “Fully Tidal”, “Fully Tidal Bayland”, and “Shallow Bay” were considered "retained."

## Results

### Data synthesis

Resulting GIS data layers (Fig 1) are available for download and exploration from the Pacific Marine and Estuarine Fish Habitat Partnership’s website (https://pacificfishhabitat.org, see also S4 File) and are also available through web-based geospatial data platforms (ArcGIS Online, DataBasin, and NOAA Digital Coast).

### Mapping maximum tidal extent

Initial drafts of the geodatabase received extensive review from local stakeholders in two informational webinars, which were particularly useful in refining the Estuary Extent (as described above). Expert reviewers from all three states confirmed that the mapping corresponded well to their local knowledge of the estuary’s spatial location. In addition, the estuary boundary was validated in 14 estuaries spanning the Pacific Coast (Fig 2), and EBEEM results were compared to locally-generated mapping of historical estuary extent in the Sacramento-San Joaquin estuary (see below).

**Fig 4:**
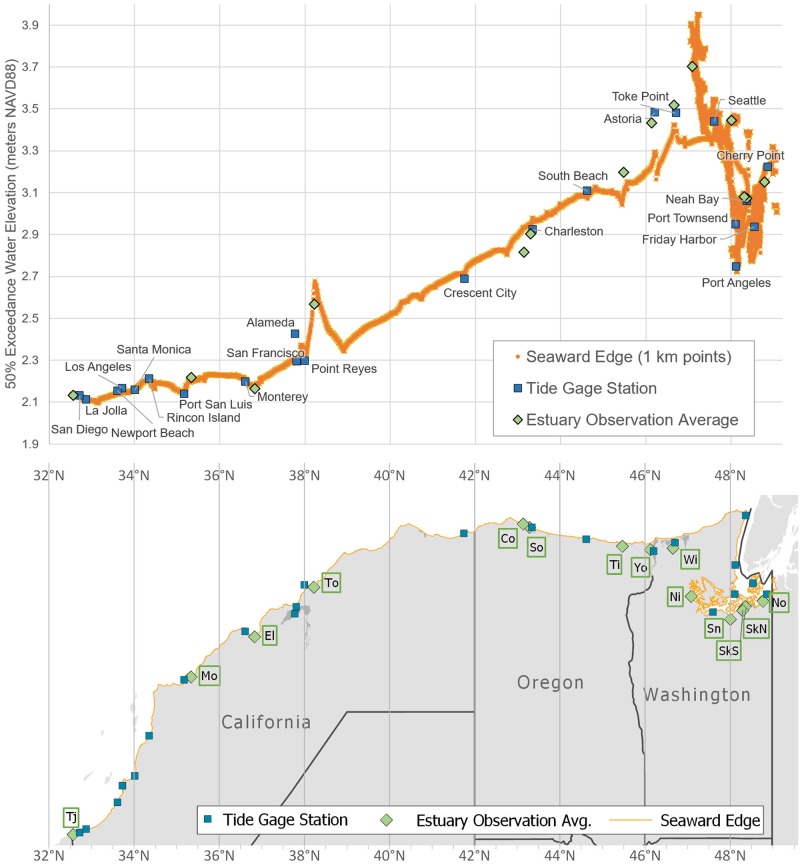
% exceedance levels on the Pacific Coast. **50** Interpolated 50% exceedance water levels (top panel) corresponding to coastal segments of the Pacific Coast (bottom panel), including the seaward edges of mapped estuaries. Also noted are NOAA’s 22 long-term tide gauges used to predict 50% exceedance contours (blue squares) and average values from 14 ground-truthed estuaries (green diamonds) shown in Fig 2. Note that 50% exceedance values within estuaries are adjusted using VDatum and therefore vary from the seaward edge values (orange line).

EBEEM’s interpolation of NOAA’s 50% exceedance values clearly shows the West Coast’s strong latitudinal clines in high water (Fig 4). This cline matches Pacific Coast observations of the latitudinal variation in tides [44]. 50% exceedance varied from 2.1 m along the coast of the Southern California Bight, increasing to up to 3.4 m along the Washington Coast. Estuaries subject to tidal amplification show higher 50% exceedance levels, a pattern most observable for southern portions of Puget Sound, which showed the highest observed 50% exceedance levels (>3.9 m) on the coast. Nevertheless, additional spikes in high water were observable along the coast, including at the mouths of San Francisco Bay, Tomales Bay, the Columbia River, and Willapa Bay. Within all 14 validation estuaries, 50% exceedance elevation was approximately 0.5 m higher than MHHW.

**Fig 5:**
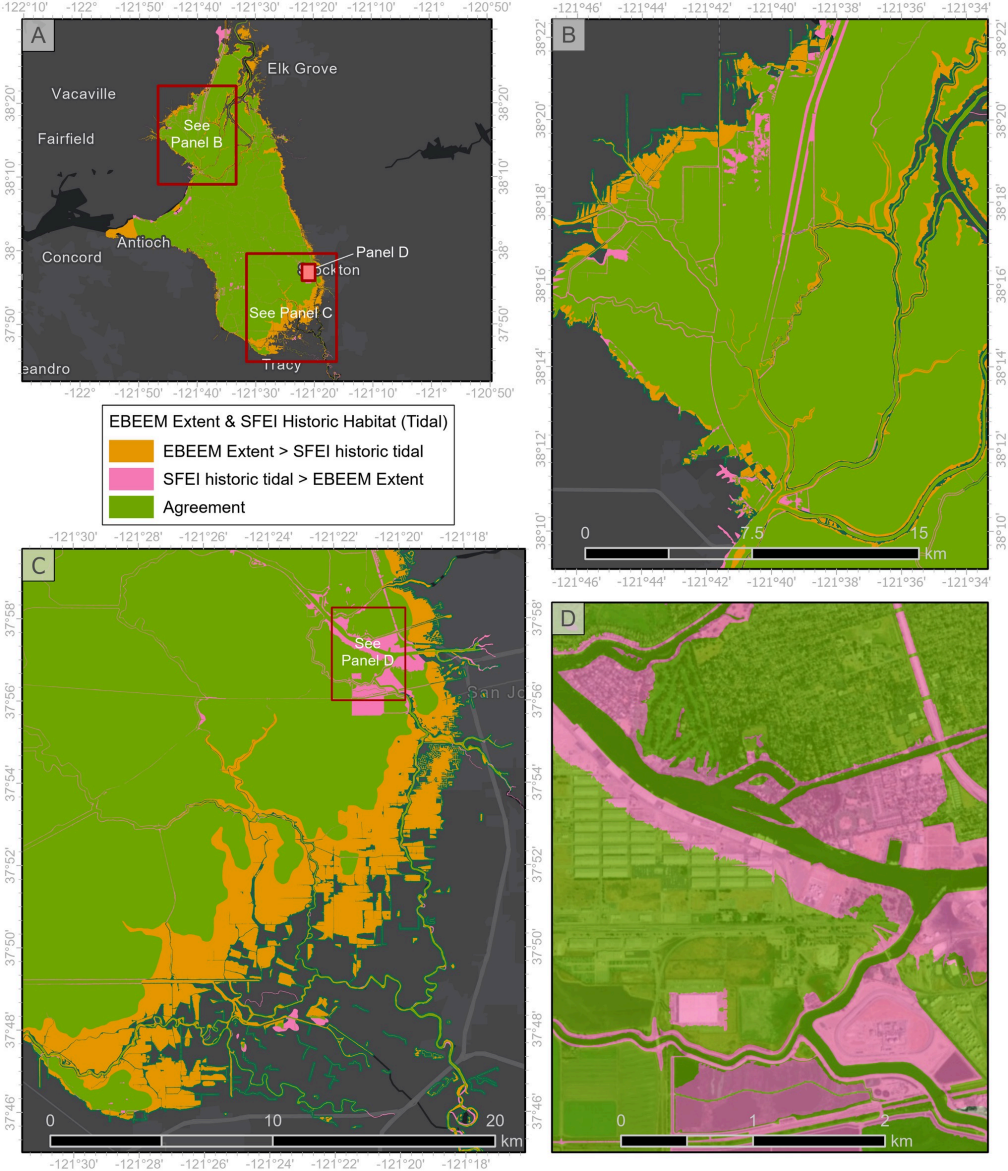
Comparison of historical estuarine footprint in the Sacramento-San Joaquin delta. The comparison illustrates agreement and disagreement between the historical footprint from EBEEM mapping (PMEP) compared to historical ecology mapping by the San Francisco Estuary Institute [17]: the entire extent of the delta (A), a magnified region in the north delta (B), a region in the south delta (C), and an urban area in the central delta with a large amount of disagreement between mapping efforts (D).

The EBEEM model closely replicated other, locally-informed historical mapping efforts. We focus on the San Francisco Estuary Institute’s (SFEI) historical mapping of the Sacramento-San Joaquin delta [17] to illustrate the degree of agreement between methods. These methods are completely independent approaches to the same problem–Whipple et al. [17] used a combination of historical maps, records, and surveys, while EBEEM maps current tidal datums. Nearby NOAA stations with EWL analyses are located near San Francisco and Alameda, outside the delta area and therefore unlikely to reflect the strong freshwater forcing present within the delta area from California’s largest river (the Sacramento). Nevertheless, we found 85.2% agreement based on the union of locally-generated maps of the historical extent of tidal wetlands [17] with EBEEM maps (Fig 5).

### Estuary identification and classification

**Fig 6:**
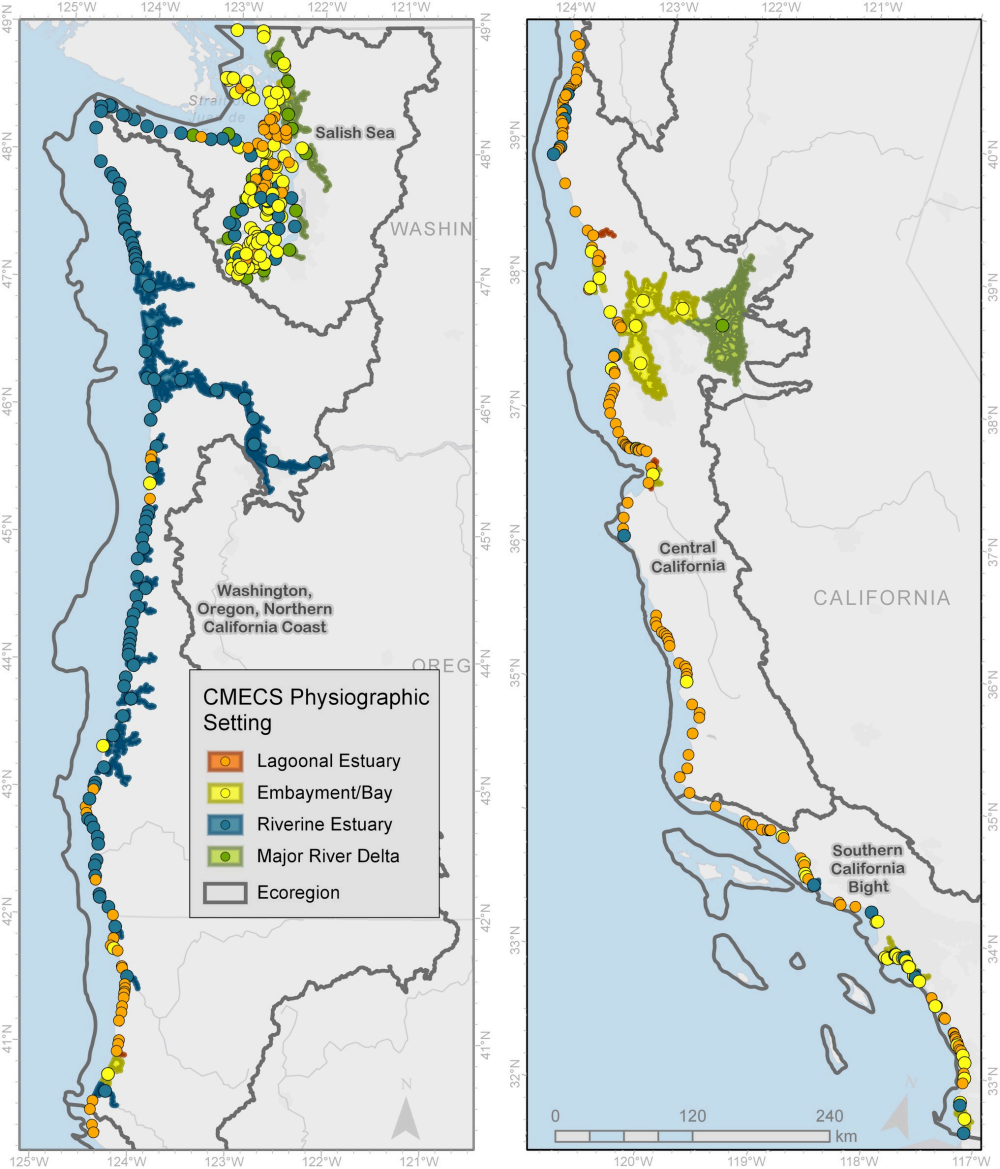
Location and physiographic type of 444 estuaries in four ecoregions of the Pacific Coast. Individual estuaries are denoted by circles of different colors corresponding to physiographic type. For larger estuaries, the entire polygon is shaded with the same colors.

We refined an initial analysis of the tri-state Pacific Coast [8], to 444 estuaries (Fig 6 and S4 File) after applying CMECS classifications of physiographic setting and geoform to areas mapped within the 50% exceedance contour (Fig 4). Collectively, these estuaries totaled 735,198 ha of historical estuary area among four marine ecoregions (Table 4): 1) the Salish Sea, 2) Washington, Oregon, and Northern California coasts, 3) Central California, and 4) Southern California Bight [45]. In three cases (San Francisco Bay, the Columbia River estuary, and the Salish Sea), larger estuaries were subdivided into smaller units, reflecting existing geomorphic and local naming conventions.

**Table 4:** Summary of estuaries identified in four ecoregions of the Central Pacific Coast of the United States. Total area is current plus historical estuary extent as defined by EBEEM mapping, and most common estuary types are abbreviations of CMECS terms: major river delta, riverine estuary, and embayment/bay.

	Salish Sea	WA, OR, and N. CA	Central CA	S. CA Bight	Total
**No. of estuaries**	166	110	107	61	444
**Total area (ha)**	81,946	252,207	380,734	20,313	735,198
**Most common estuary type and proportion of total area**	Delta 0.694	Riverine 0.938	Embayment 0.535	Embayment 0.841	

**Fig 7:**
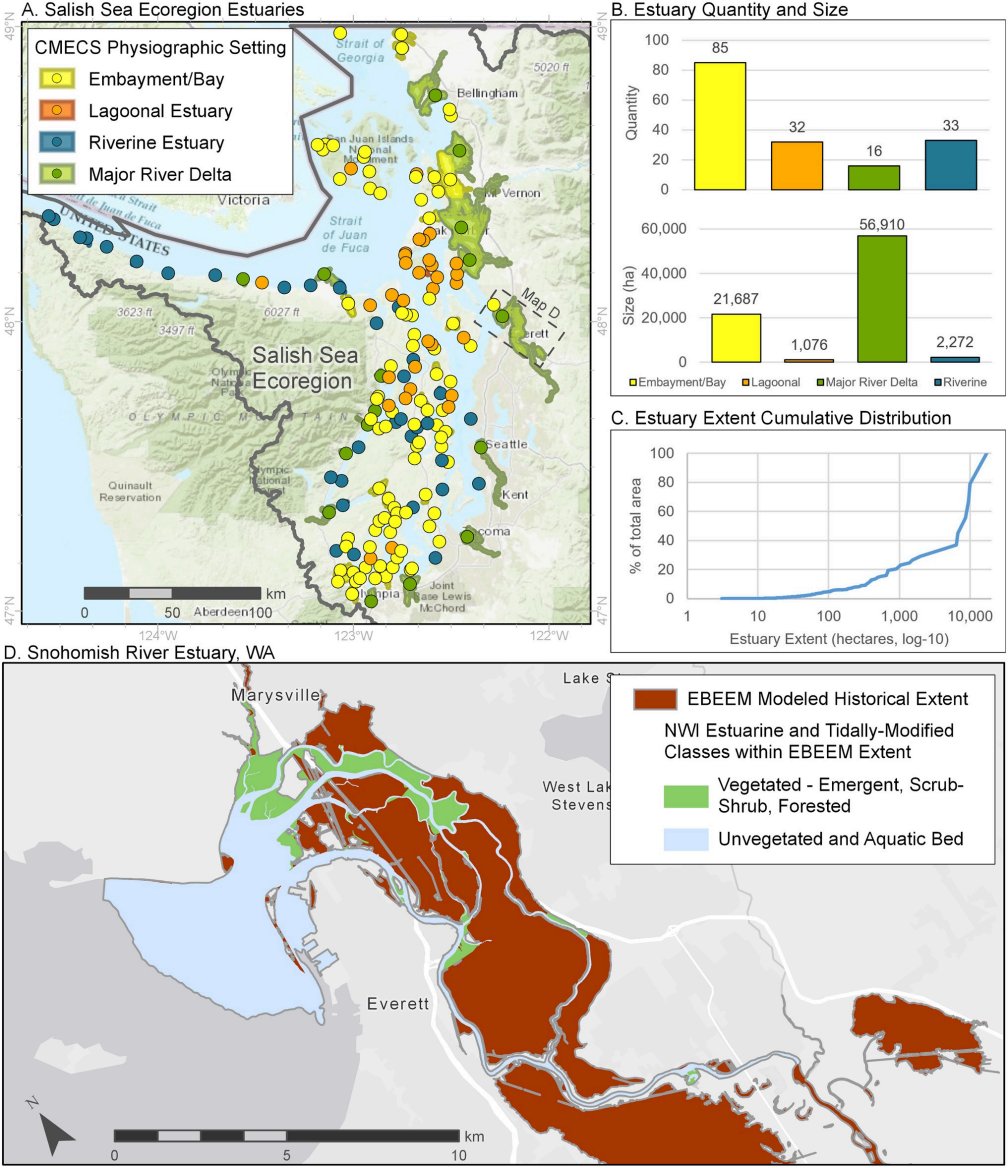
Summary of estuaries in the Salish Sea/WA coast ecoregion of the Pacific Coast (Washington, Oregon and California). Map in upper left panel (A) shows the number and extents of estuaries by physiographic type. Upper right panel (B) shows the extent percent representation of each estuary type as calculated by the number of each type (Quantity) and by areal coverage (Size), while the middle right panel (C) illustrates the cumulative distribution function of the number of estuaries as a function of their size in hectares (ha). The map in the lower panel (D) illustrates an example estuary from each the ecoregion including spatial extent of historical footprint based on 50% exceedance contour (“EBEEM”) and area of current tidal wetlands based on National Wetland Inventory classes (“NWI”), data used to calculate habitat loss. Note: Unvegetated and aquatic bed areas shown in blue are within the EBEEM Historical extent, but were excluded from the wetlands loss analysis.

The Salish Sea comprised 166 estuary units (Fig 7). While embayments were the most common physiographic type (85 estuaries), lagoons were numerically abundant within Puget Sound and riverine estuaries were more common along the Strait of Juan de Fuca. However, 16 major river deltas accounted for ~70% of the total estuarine area for this region. These are prograding deltas extending into the deep basins of Puget Sound (Fig 7D). Across all physiographic types, the cumulative distribution of estuary size is strongly shaped by systems larger than ~8,000 ha.

**Fig 8:**
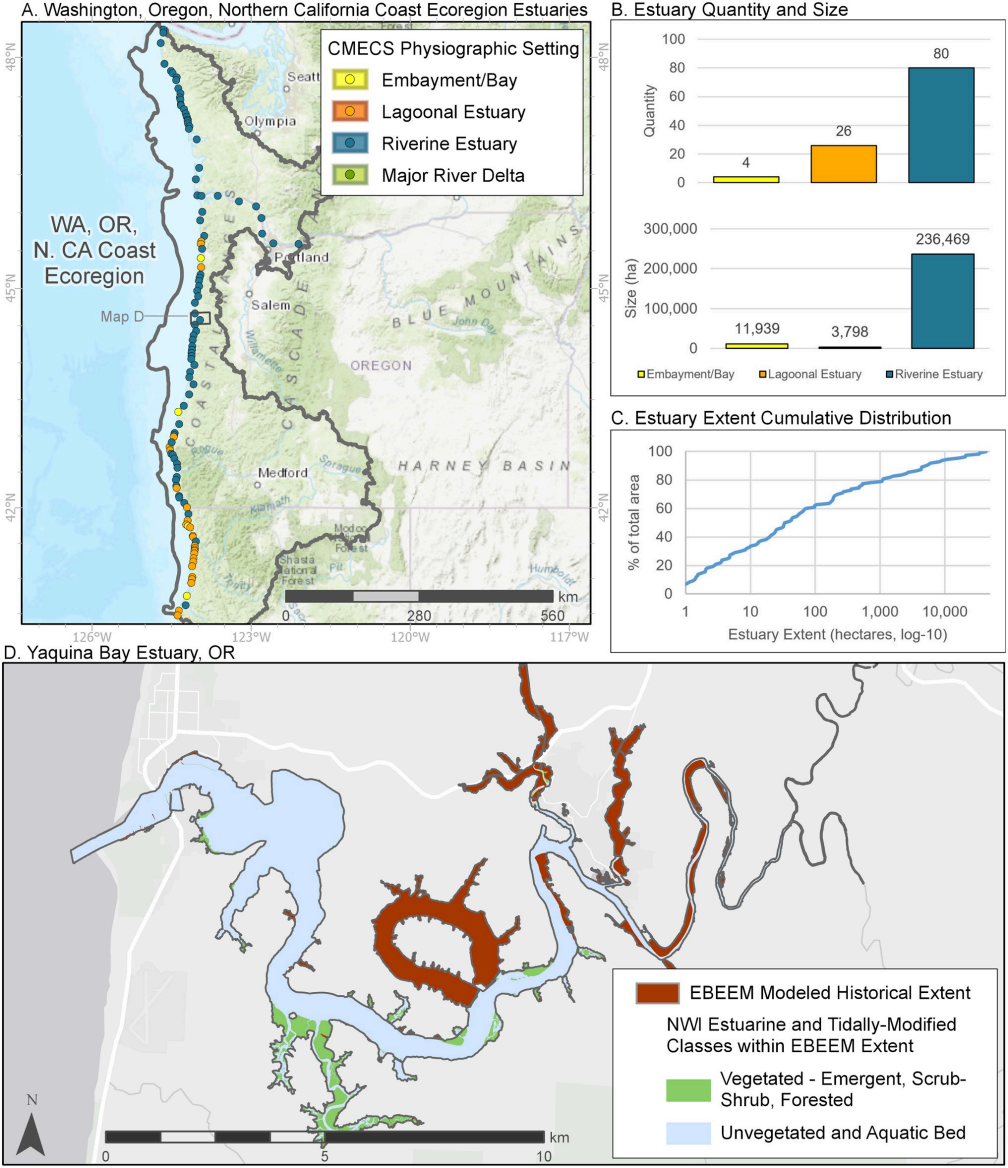
Summary of estuaries in the Oregon and Northern California ecoregion of the Pacific Coast. See Fig 7 for details.

The 110 estuaries of the Washington, Oregon, and Northern California coasts (Fig 8), were dominated by riverine estuaries both in number (80 estuaries) and area (~94%). Lagoons (26 estuaries), the next most common physiographic type, were found mostly in California. The similarity of type as well as the lower variation in size of river systems entering this ecoregion resulted in a much more continuous cumulative distribution of individual estuary area, although several large estuaries nevertheless created a log-normal size distribution. Many of these riverine systems in this region are incised, resulting in the EBEEM-mapped historical estuary extending much farther upriver (Fig 8D) than has been previously acknowledged [46, 47].

**Fig 9:**
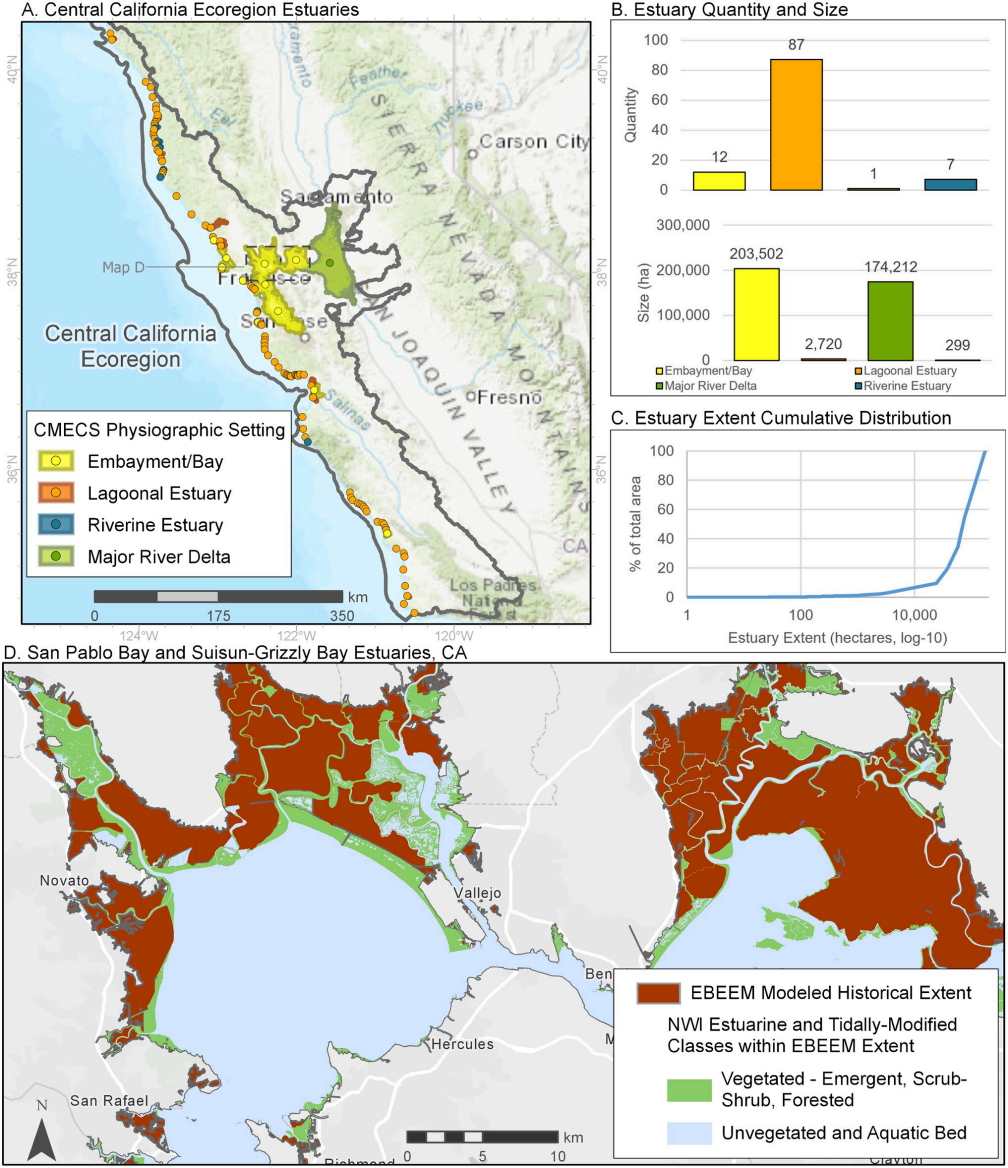
Summary of estuaries in the Central California ecoregion of the Pacific Coast. See Fig 7 for details.

Within the Central California ecoregion (Fig 9), 87 of the 107 identified estuaries were coastal lagoons. However, these small systems collectively account for less than 1% of the region’s historical wetland area, overshadowed by the Sacramento San Joaquin delta (~46% of area) and connected embayments within greater San Francisco Bay (53% of area). These latter systems created a highly skewed distribution of historical estuary area. Most of the historical wetlands of the Sacramento San Joaquin delta and San Francisco Bay have been diked and converted to agriculture and other human uses (Fig 9D).

**Fig 10:**
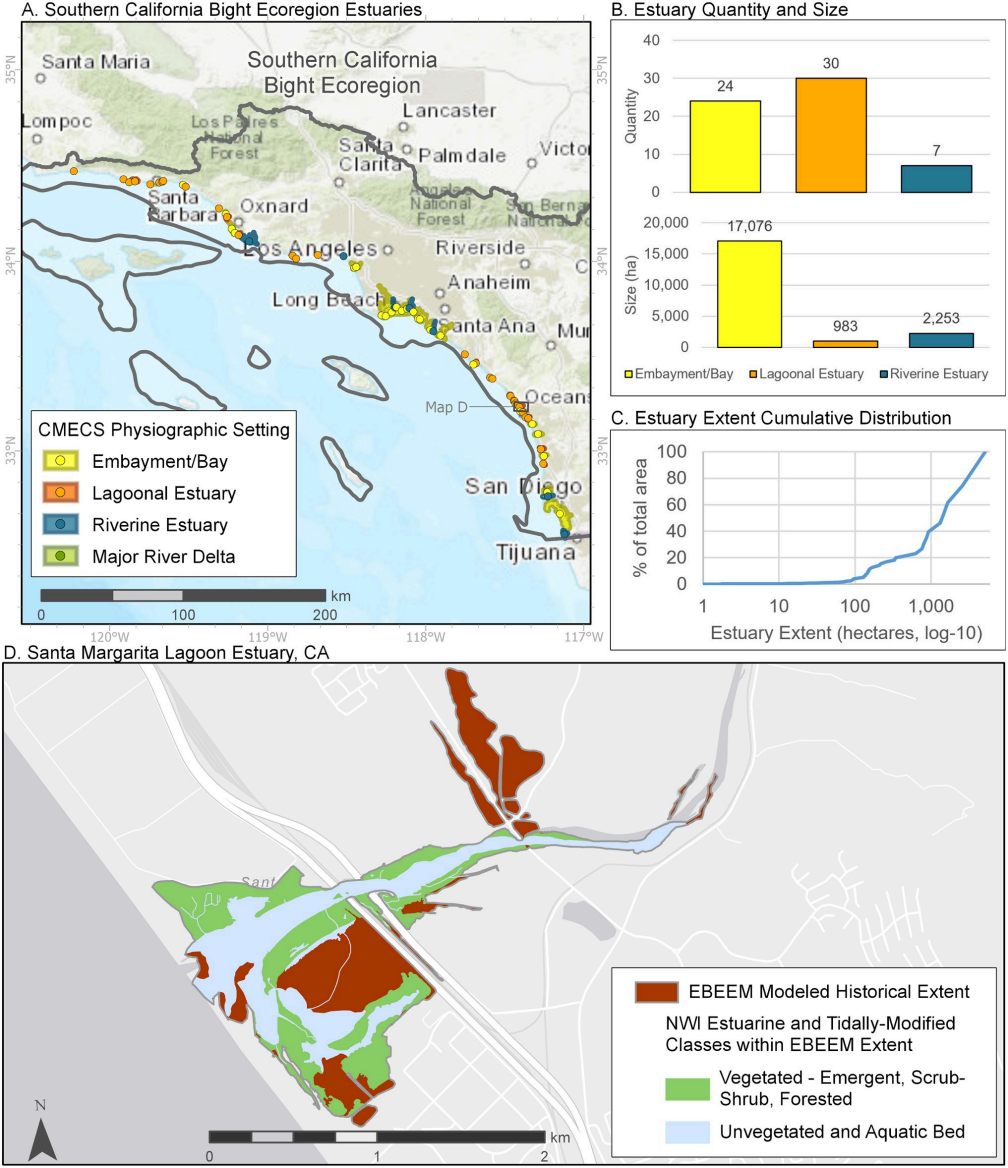
Summary of estuaries in the Southern California Bight ecoregion of the Pacific Coast. See Fig 7 for details.

Like estuaries in Central California, estuaries in the Southern California Bight (61 estuaries, Fig 10) are composed primarily of lagoons, yet several large embayments account for 84% of the historical estuarine wetland area. This pattern likewise resulted in a skewed distribution of estuary sizes. Many of these, like the Santa Margarita estuary illustrated in Fig 10D, have been subject to a large amount of fill. Therefore, historical estimates of wetland footprint in this region are especially sensitive to anthropogenic alterations in lidar elevations, resulting in the EBEEM mapping likely underrepresenting actual historical estuary area. These alterations are easily observable as straight boundaries or as roadways through units (Fig 10D).

### How much estuarine wetland has been lost?

**Fig 11:**
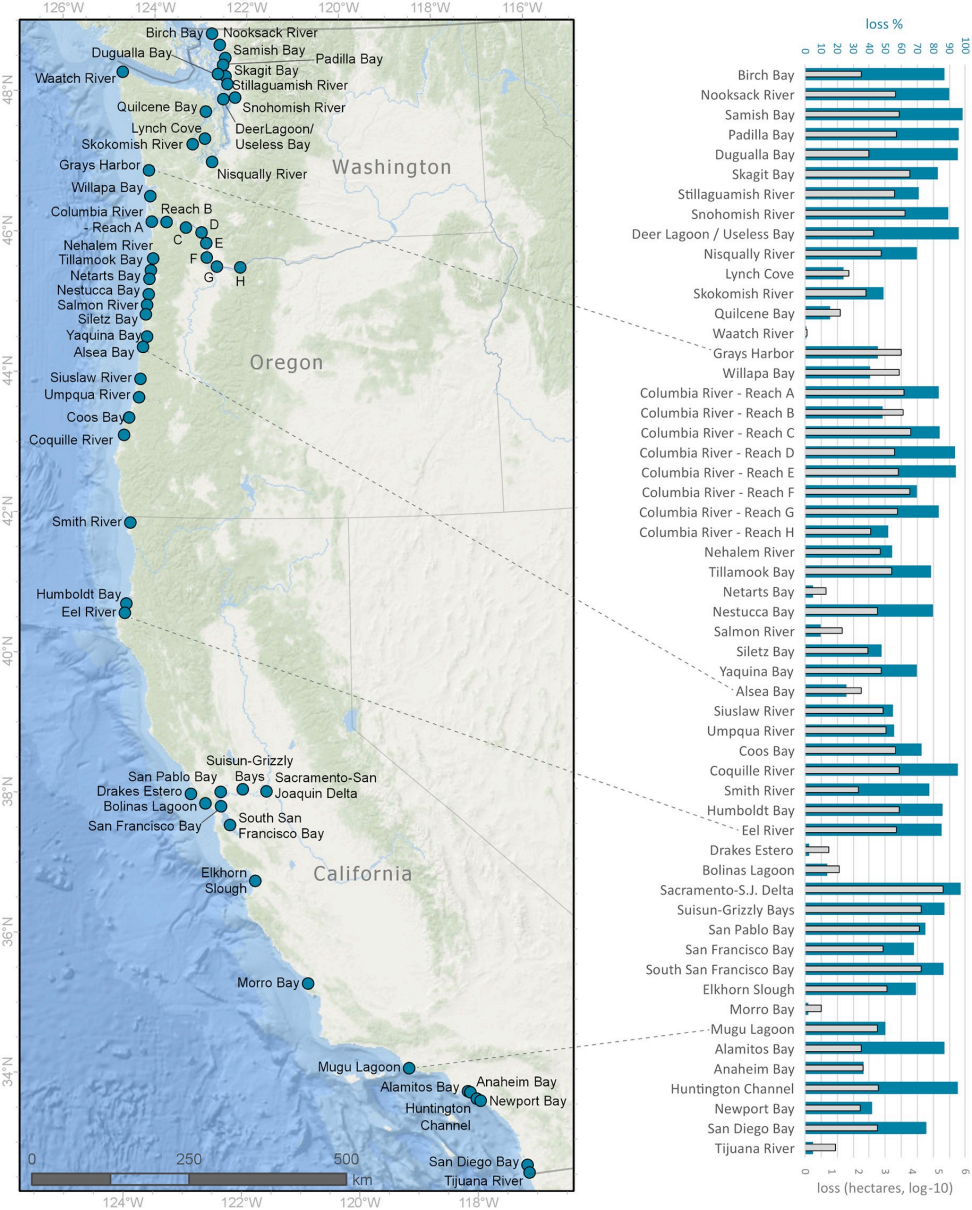
Wetland loss in 55 estuary systems across the Pacific Coast. The map at left denotes all estuaries used in the analysis, and the bar graph at right illustrates both % estuarine wetland loss (blue bars, top axis) and amount of loss in hectares (gray bars, bottom axis), based on indirect assessment (comparison of NWI data with EBEEM).

Across the 55 estuaries analyzed, overall tidal wetland loss was 85%. Loss by individual estuary varied greatly (1–98%) (Fig 11). Twenty-eight estuaries were estimated to have undergone more than 70% tidal wetland loss; conversely only 10 estuaries had less than 30% loss. Of the three estuarine types analyzed, major river deltas were the most impacted with 94.9% overall loss of tidal wetlands (Table 5). The other two estuary types also had large overall losses: 82.0% for embayments/bays and 67.1% for riverine estuaries. Significant wetland loss (approximately 60 to 90%) has occurred within all ecoregions (Table 5). Due to urbanization, the Southern California Bight estimate (59%) probably underrepresents actual losses.

**Table 5:** Area and percent loss of tidal wetlands in emergent, scrub-shrub and forested classes for 55 estuaries on the Pacific Coast, by estuary type and marine ecoregion.

	# of estuaries	Tidal wetland loss (ha)	Historical tidal wetland area (ha)	% loss
**Ecoregion**				
Salish Sea	13	25,931	30,448	85.2
WA, OR, N. CA	26	60,107	88,164	68.2
Central CA	9	213,882	233,271	91.7
S. CA Bight	7	1,965	3,347	58.7
**Estuary type**				
Embayment/Bay	20	72,865	88,870	82.0
Major river delta	9	171,662	180,856	94.9
Riverine estuary	26	57,358	85,505	67.1
**Total**	55	301,885	335,230	85.0

We compared our indirect assessment of wetland loss with three local mapping efforts that directly mapped wetlands lost to diking and other barriers. In each of these three efforts, the EBEEM-based indirect assessment of estuarine wetland loss performed similarly to the local assessment, with agreement ranging between 82% and 95% (Table 6). In each set of estuary units examined, most areas of disagreement occurred where the indirect assessment identified wetland loss, but the direct assessment indicated retained wetland. These findings suggest that our indirect method may slightly over-estimate wetland loss, except in areas of significant fill. Nevertheless, the high level of agreement in these comparisons suggests our method is both accurate and applicable to very large geographies of multiple estuary systems. Spatial differences between the datasets highlight areas that may need NWI updates or further investigation of tidal connectivity.

**Table 6:** Comparison between this project’s region-wide tidal wetland loss assessment (Region) and local assessment (Local) for the Lower Columbia River estuary [41], San Francisco Bay [42], and Oregon estuaries (http://www.coastalatlas.net/cmecs).

Estuary system(s):	Columbia River		San Francisco Bay		Oregon Coastal estuaries	
Determination	Area (ha)	%	Area (ha)	%	Area (ha)	%
Agree: lost	21,817	67.2	59,261	80.0	17,220	53.3
Agree: retained	8,552	26.4	11,505	15.5	9,357	28.9
Disagree: Region = lost, Local = retained	1,849	5.7	1,764	2.4	5,342	16.5
Disagree: Region = retained, Local = lost	232	0.7	1,507	2.0	414	1.3
Not classified in Local	6,723		0		0	
Total area (agree + disagree)	32,449		74,036		32,333	
Total agreement	30,369	**93.6**	70,766	**95.6**	26,577	**82.2**

## Discussion

Because tidal wetlands are defined by predictable inundation frequency, a logical way to map tidal wetlands is via water level models. The EBEEM method produces maps of tidal extent that closely match previous detailed estuary-specific findings of historical tidal wetland footprints. This approach does not replace system-specific efforts relying on other datasets such as historical maps and T-sheets. Rather, our method provides a straightforward and accurate way to efficiently map the historical estuarine footprint for large spatial extents using readily available datasets: tide gauges, datum adjustment tools (e.g. VDatum), and high-resolution elevation data. Once EBEEM mapping has been completed, estimates of wetland loss are possible for areas with data on current estuarine wetland extent. We demonstrate this with the use of NWI maps, although other data could be used. We illustrate the power of EBEEM by estimating wetland loss for 55 Pacific coast estuaries. These indirect estimates of loss compare favorably with more direct efforts to estimate estuarine wetland loss, again validating this approach and its applicability for large spatial extents.

### Characterizing maximum tidal wetland extent

Our mapping begins from a clear and standardized definition of estuarine systems: areas subject to tidal influence, extending upslope from the sea to topographic surfaces inundated by the highest annual tides. Our ground-truthing efforts and comparisons to intensive local scale mapping showed this approach to be accurate and representative of the inland estuarine extent. Given the wide variety of estuary types and tidal wetlands on the West Coast, the EBEEM method is broadly applicable for mapping the estuary’s upslope boundary.

The EBEEM model extends extreme water levels across the coast, illustrating a latitudinal gradient in the 50% exceedance elevation among the 22 long-term gauges. Significant local variation from the latitudinal trend exists, particularly in areas with high degrees of tidal amplification such as Puget Sound. Also intriguing were a few spikes in 50% exceedance level along the coast, such as that associated with Tomales Bay. Because spikes like this were not associated with high values at the most local gauges and the Estuary Extent was validated by ground-truthing, it is possible that coastal spikes are the consequence of either real increases in 50% exceedance due to local forcing from currents, winds, or tidal amplification [48], or bias in local lidar DEMs, resulting in inaccuracies in the VDatum model.

Ground-truthing of fourteen estuaries along the Pacific coast supports the idea that the 50% exceedance interval represents a good demarcation of regular tidal inundation (Fig 2). Additional ground-truthing in Oregon [49–51] suggests that most areas near the 50% exceedance contour actually experience tidal inundation much more often than every two years, and are in fact inundated multiple times per year. This apparent discrepancy is probably due to two main factors. First, the lidar DEM in Oregon is typically elevated (biased upwards) by 10–30 cm above the actual ground surface due to vegetation interference [52, 53], and such bias is probably also present in Washington and California. Second, river (fluvial) inputs to estuaries or storm surges can substantially increase inundation above predicted high tides during the rainy season [47, 48, 54, 55]. The NOAA tide gauges that provide EWL analyses are generally located near the mouths of rivers, where the “fluvial effect” is not great, but the effect increases up-estuary as the river valley becomes more confined, amplifying the fluvial signal [48, 54, 55].

The effectiveness of using the 50% exceedance elevation as the estuary boundary was supported by comparisons with other historical mapping efforts such as San Francisco Bay (Fig 5). This comparison indicated a high correspondence (85%) in estimation of historical extent, despite using very different methodologies. These historical ecology efforts are quite intensive, with much scrutiny of local detail, and are therefore restricted to smaller geographic extents. The high level of agreement between these highly detailed methods and our simple approach supports the utility of our method, since it can be efficiently applied to large geographies and produce accurate results.

### Benefits of new mapping

Our method provides an opportunity for documenting historical estuarine footprints and wetland loss across large spatial extents. Because historical estuary footprints are often unknown or estimated from relatively recent baselines [10, 11], it is often difficult to define total estuarine wetland loss. This issue is compounded when the full extent of freshwater tidal zones is not included. Hence, estuary definitions based solely on salinity gradients [56] or elevations subject to daily tidal inundation [27], or administrative boundaries that consider all freshwater wetlands as fluvial, will underestimate tidal wetland loss. Increasing recognition of the importance of freshwater tidal areas as buffers from flooding [57, 58] transitional rearing habitat for diadromous fish [59, 60], and future sites of brackish waters in response to sea level rise [58] raises the importance of better quantification of freshwater tidal portions of the estuary. Our levation-based method provides a new tool toto improve the mapping of these areas. Elevation-based mapping also greatly improves the mapping of brackish scrub-shrub and forested tidal wetlands, the extent of which has been greatly underestimated in the past in NWI and other comprehensive mapping products because these wetland classes may not be easily recognizable as tidal wetlands, compared to tidal marsh.

Our efforts also reveal that definitions of maximum estuary extent deserve critical review. For example, definitions within CMECS for maximum extent which use MHHW or "supratidal" in the Estuarine Coastal subsystem likely result in a significant underestimate of estuary extent. Although Mean High Water of Spring Tides may be a reasonable approximation for all intertidal wetlands (whether Estuarine Coastal or Estuarine Tidal Riverine Coastal), this datum is not universally available and may be too low given the upwards elevation bias of lidar DEMs, which are likely to be used as the basis for mapping in many estuary systems. Our method provides a relatively simple solution for utilizing lidar to extend estuary boundaries to their proper landward position. The benefits of EBEEM can apply to classifications within NWI as well, as revised estuary extents can be used to identify areas potentially needing updates or corrections in NWI.

The applications of extending estuary boundaries upstream are multifold. A primary result for the Pacific Coast (e.g., Figs 7–10) and an implication for other regions is that in some cases, estuarine footprints need to be extended upriver. These expanded footprints help us better understand the true extent of our estuaries as defined by tidal inundation across all seasons of the year. Hence, these footprints will likely better highlight inundation risk [61] or salinity intrusion [62], since mapping 50% exceedance probabilities will reveal areas likely to be subject to regular *tidal* flooding and therefore more prone to inundation during extreme tides and storm surges. Thus, mapping 50% exceedance provides a better baseline than mean lower low water or other tidal datums for projecting increased flooding due to sea level rise [63, 64], and likely holds promise for better projection of areas amenable to carbon storage [65]. Where habitats are being restored for estuarine-dependent species [66–68], elevation-based models will be an important tool to identify locations on the landscape where restored wetlands will likely be maintained by natural habitat-forming processes.

The combination of comprehensive inventorying of estuaries with EBEEM mapping across such a large and variable area illuminated patterns important to regional management and conservation. First, 55 of the 572 estuaries inventoried account for 97% of historical West Coast vegetated tidal wetland habitat. Our indirect loss assessment found that 85% (301,885 ha) of vegetated tidal wetlands have been lost from these larger systems. This alarming amount of loss highlights the critical need to maintain and expand upon the large extents of estuarine habitat in larger estuaries. These habitats are crucial to the many rare and endangered species as well as migratory birds along the Pacific Flyway, which rely on these productive feeding and resting grounds. Secondly, while of less cumulative area, smaller lagoonal systems are numerical dominants in areas such as Southern and Central California, and within Puget Sound. While these smaller systems do not contribute large areas to the region, their individual and collective contributions may still be regionally important. For example, research has shown fish such as salmonids move among these smaller estuaries for beneficial growth and survival [60, 69, 70], and migratory birds may benefit from smaller dispersed estuarine resting and feeding habitats [71], reinforcing the need to consider these smaller systems in regional restoration and management goals.

### Challenges and uncertainties in estimating estuary extent

Despite the benefits of EBEEM, we note that there are certain circumstances in which EBEEM will likely perform poorly and may not accurately estimate the historical estuarine footprint. These situations depend both on data inputs as well as predictions from the model.

#### Data inputs

As EBEEM relies on lidar datasets, it is susceptible to inherent inaccuracies of lidar DEMs. For example, vegetation interferes with transmission and return of the lidar signal, generally resulting in an upward bias in the DEM. Lidar data processing algorithms used during construction of the DEM aim to reduce or eliminate vegetation interference; nonetheless, upward bias of the lidar DEM in typical Pacific Northwest tidal marsh vegetation typically ranges from 10 to 30 cm, but can be as high as 50 cm in some vegetation types [52, 53]. This upward bias can potentially be corrected through post-processing to adjust the DEM using other types of remote sensing data or field surveys [53, 72, 73]. However, such field-based corrections are not possible across a very large study area such as the entire U.S. West Coast. To achieve accurate results despite this challenge, the EBEEM method was developed with vegetation interference in mind. That is, we based our choice of the 50% exceedance datum on field observations of tidal wetland locations, and then determined their elevations using the lidar DEM. Hence, the EBEEM boundary already accounts for typical vegetation interference. Nevertheless, some vegetation types (e.g., slough sedge (*Carex obnupta*), Lyngbye’s sedge (*Carex lyngbyei*)) can cause a particularly large upward bias in the lidar DEM [52], which may have reduced the EBEEM footprint in certain areas, resulting in omission of historical or current tidal wetlands from the EBEEM mapping.

More importantly, the age of lidar data can affect the accuracy of the DEMs. Changes in topography that occurred after the date of lidar data collection will not be reflected in the DEM, leading to inaccuracies in EBEEM mapping. For example, restoration areas excavated from higher ground may be omitted from the EBEEM mapping if the excavation occurred after lidar data acquisition. Similarly, lands filled after lidar data acquisition may be included in the EBEEM mapping even if they are now above tide range. Such changes, while generally small in area, nevertheless may limit application of this method in some estuaries where such conditions are prevalent. Systematic efforts to update lidar as restoration or filling occurs will improve the utility of our approach.

#### Model prediction inaccuracies

EBEEM relies on both interpolation and extrapolation from locations where 50% exceedance has been observed. NOAA provides EWL data for only 22 stations along the central Pacific Coast (Fig 2, [32]). Therefore, to map the estuaries between these stations, we spatially interpolated 50% exceedance interval among gauges provided by NOAA. Furthermore, we extrapolated exceedance contours into estuaries landward of VDatum coverage. Of these two types of predictions, interpolation likely results in fewer inaccuracies, due to continuous VDatum coverage and steep elevational gradients along the Pacific Coast.

Landward extrapolation is more challenging because of both tidal and fluvial dynamics and can affect predictions for both lagoonal and riverine estuaries. Lagoonal estuaries may experience their highest water levels during periods of estuary mouth closure rather than during high tide events [41], so prediction of 50% exceedance depends less upon tidal inundation and more upon the temporal interaction of estuary closure and the freshwater hydrograph. In riverine estuaries, the fluvial component of the inundation regime, which generally increases landward in estuaries [48, 54, 55] can be substantial [31, 74]; but this fluvial component is not reflected in NOAA EWL models for outer coast stations. In addition, the natural elevation gradient of the river surface is not reflected in outer coast EWLs, and this gradient can be a major factor for large rivers such as the Columbia. We used locally-generated EWL values for the Columbia (see Columbia River Estuary above), but other rivers may be affected by this uncertainty to a lesser extent. For optimal results, an elevation-based method like EBEEM would incorporate hydrodynamic models to generate predicted extreme water levels and recurrence intervals. Such modeling has been developed for the larger more intensively studied estuaries of the West Coast such as San Francisco Bay [75], the Lower Columbia River [76] and Puget Sound [77], but is not available for most of our large West Coast study area. In any case, inaccuracies in predictions of tidal inundation within fluvially dominated portions of estuaries exist for these models as well [78].

Landward extrapolation is particularly challenging in areas that have undergone extensive anthropogenic modification. Urbanized estuaries may contain substantial areas that have been artificially filled above the 50% exceedance elevation, resulting in a large portion of the historically inundated area now located above the 50% exceedance contour. Similar issues can affect less-urbanized estuaries where localized fill (e.g., road embankments) fragments the historical estuary footprint. In both cases, the result is underestimation of historical estuary extent (and underestimation of estuarine wetland losses). We observed evidence of both problems in our EBEEM maps; for example, in many urbanized estuaries of the Southern California Bight, the historical extent of the estuary as mapped by SFEI [16] is larger than the EBEEM mapping due to substantial areas filled above the 50% exceedance elevation. In both situations, historical maps such as SFEI’s (based on data other than current elevations) can help locate areas that were historically tidal wetlands.

Conversely, wetlands with high soil organic content often subside (i.e., their surface elevation drops) upon drainage [79–81]. Drainage of nontidal wetlands adjacent to the historic tidal wetland boundary could have caused enough subsidence to bring these areas into tidal range. If so, these areas will be mapped in EBEEM but may not be part of the historical estuary. Possible examples are found along the fringes of the Sacramento/San Joaquin River delta, in which agricultural lands have undergone subsidence of 3 to 5 m or more [82, 83]. Nonetheless, such areas are likely to be either current tidal wetlands, or restorable to tidal wetlands, so we view this source of error as less important compared to filled areas.

### Challenges in inferring wetland loss

Due to the spatial extent of the analysis and lack of data to support direct assessment, this study’s loss assessment uses an indirect method for identifying wetland loss. This method uses wetland data from NWI, which has its own limitations and challenges. Among these are the NWI’s failure to map some tidally influenced wetlands, particularly in upper estuaries and in forested tidal wetlands that lack visual indicators of tidal influence [84–86]. Other limitations of the NWI for this assessment include the age of NWI data [85] and changes to wetland footprint post-restoration, and the scale of the NWI data (1:24000), resulting in inadequate mapping of small features [85] and consequently greater impact of classification errors in smaller (<100 ha) estuaries. Similarly, NWI does not accurately map subtidal and low intertidal vegetated wetlands such as seagrasses and algae; and because these habitats are not usually converted to non-tidal lands when altered or lost, the method cannot estimate loss of such habitats. Further, lagoonal estuaries were not included in the loss assessment because—even if undisturbed—areas within the estuary extent for lagoonal systems would not necessarily be coded as tidal in the NWI, so alterations and losses could not be evaluated using this method. Despite these challenges, our indirect method performed well (80–95% accuracy) for a diverse set of estuaries on the Pacific Coast when compared against direct methodologies (Table 6).

Direct assessment of tidal wetland losses using high quality mapping of disconnected areas would eliminate most of the challenges and uncertainties for the indirect loss assessment, and would generate more accurate data. Therefore, a major data improvement for understanding estuarine wetland status would be spatially-explicit mapping of hydrologically-disconnected areas. Although tidal flow barriers such as dikes and tide gates have been mapped in several areas [87, 88], mapping of land areas affected by such barriers is not a simple task. In the very flat topography of former tidal wetlands, hydrologic connections, flow paths and flow direction are complex and not easily mapped, particularly when artificial barriers reroute flows [89, 90]. Development and consistent application of effective analytical techniques to directly map disconnected former tidal wetlands within the EBEEM mapping would provide a vital resource for accurate assessment of losses and for planning and prioritizing restoration actions.

Our indirect assessment of tidal wetland loss relied on NWI data to identify tidal and non-tidal areas. Due to the age of the NWI data, many restored tidal wetlands were still attributed as non-tidal in the NWI, and were therefore classified as “lost.” To reduce this source of error, we recommend updating the NWI where needed. To support such updates, we recommend mapping restored areas. Surprisingly, no comprehensive datasets showing the spatial extent of restored areas are available for the West Coast. Spatial datasets of restoration sites exist [91, 92] but these often consist of point features or rough outlines; for accuracy, these should use elevation-based mapping to determine the areas where tidal inundation has been restored. Each restoration area should also be attributed with information about the types of restoration conducted—necessary to distinguish between projects where tidal flows were fully restored (e.g. dike and tide gate removal), partially restored (e.g. use of self-regulating tide gates that allow partial tidal exchange), or not restored (e.g. riparian plantings within diked sites). An example of such a mapping project is EcoAtlas (https://ptrack.ecoatlas.org/), wherein polygons within restored areas in California are attributed with wetland and other information.

### Usage recommendations

The EBEEM mapping is useful for understanding the historical extent of the estuary, subject to the challenges and uncertainties described above. This mapping is especially useful for landscape-scale understanding and broad planning efforts, such as strategic plans, recovery plans, and coast-wide prioritizations.

Examples of the use of EBEEM data include a baseline for understanding past changes in estuary extent due to alterations, and potential future changes from land use stressors or due to climate change and associated sea level rise. An elevation-based map of estuary extent provides a powerful foundation for such analyses, which in the past have often been based on less accurate data. Elevation-based mapping of historical estuary extent [35] and analysis of historical wetland mapping [93] are being used for restoration planning and prioritization in the Lower Columbia River Estuary [94, 95]. In the same way, the EBEEM mapping can be used as a basis for local and regional determination of estuarine wetland losses, and establishment of conservation and restoration priorities, for other areas. This study’s indirect assessment of tidal wetland losses may serve as a preliminary guide to such priorities, which can then be refined using local data.

The results of this analysis should also improve analysis of impacts from sea level rise. These analyses require a baseline of current tidal wetlands for determination of future change [96]. The EBEEM mapping is an ideal starting point for such analyses, because it effectively determines current areas that would be tidally inundated (if not hydrologically disconnected). Models that assess potential sea level rise impacts to tidal wetlands benefit from elevation and spatial data on hydrologically-disconnected (diked) areas [97, 98]. For this purpose, direct mapping of disconnected areas (rather than our indirect assessment of tidal wetland loss) is recommended. The EBEEM mapping is a suitable base layer for mapping such disconnected areas; used in conjunction with mapped flow barriers, the EBEEM mapping can be used to identify the former tidal wetlands affected by the barriers.

By improving our understanding of the historical extent of estuaries, use of an EBEEM baseline also improves understanding of potential future SLR impacts on cumulative wetland losses. For example, a potential 40% loss of current tidal wetland area due to future SLR may not seem extreme. However, if EBEEM mapping shows that current tidal wetland area is only 10% of the historical area, we can understand that the combination of historic losses and SLR impacts would reduce tidal wetland area to 6% of its historic level. Furthermore, EBEEM mapping can be combined with data on the elevation of wetland types and current and projected salinity regimes to identify areas where wetlands are most likely to be subject to landward migration [96, 99,100], as well as anthropogenic barriers likely to impede this process [99]. These perspectives are important in conservation planning and can provide critical insight into regional prioritization. Furthermore, specific impacts to estuary-dependent flora and fauna, also often imperiled, can then be evaluated accordingly.

Our indirect assessment of tidal wetland loss is intended only for loss comparisons at broad scales (e.g. across estuaries and regions). The loss mapping is not intended for use in a regulatory context, nor should it be used for site-specific analysis. Due to the assessment methods and data sources (see Challenges and uncertainties above), mapped areas of loss and retention are preliminary and should be locally refined using field investigation. Local refinement of the mapping is especially needed in the middle and upper estuaries of the Pacific Northwest, where the NWI often misclassifies wooded tidal wetlands as non-tidal [84]; these areas are therefore shown as “lost” in the mapping, but are in fact important remnants of high-priority, least-disturbed tidal wetlands.

## Conclusion

Rapid development pressure from increasing human populations has led to the problem of a shifting ecological baseline in estuarine wetlands, wherein standard methods using relatively recent references under-represent the true extent of wetland loss. The EBEEM method provides a way to accurately estimate historical tidally influenced footprints, and can be used in conjunction with databases of current wetland extent to infer tidal wetland loss. Furthermore, EBEEM can inform where prime estuary restoration opportunities exist. Looking forward, EBEEM mapping will be important to determine which areas are most vulnerable to sea level rise. In conjunction with current wetland information, EBEEM can be used to improve projections about what wetland types are likely to transition to other types (e.g., scrub-shrub to estuarine emergent marsh). Despite mapping challenges inherent in all large-scale mapping efforts, the EBEEM method represents a major advance in accuracy and scalability compared to past methods for mapping estuaries. We therefore expect this effort to be useful for broad understanding of estuarine wetland extent in the past, present, and future.

## Supporting information

S1 File Lidar and DEM data sources. (PDF)

S2 File Groundtruthing. (PDF)

S3 File Geoprocessing steps for indirect assessment of West Coast USA tidal wetland loss. (PDF)

S4 File Summary and locations of 444 Pacific Coast estuaries. (PDF)
