# Supplementary material for: Insights into estuary habitat loss in the western United States using a new method for mapping maximum extent of tidal wetlands
Source: PLoS One. 2019 Aug 14;14(8):e0218558. doi: 10.1371/journal.pone.0218558 (PMC6693690; doi:10.1371/journal.pone.0218558)
Supplement: S1 File — (PDF) [file pone.0218558.s001.pdf]

## S1. Lidar and DEM data sources

We compiled lidar and DEM products from a number of sources. These were put together to create a full lidar map of the Pacific Coast in Washington, Oregon, and California.

Coastwide

· NOAA SLR data (http://csc.noaa.gov/slr/)

Washington

· Puget Sound lidar Consortium (http://pugetsoundlidar.org/) for Olympic Peninsula

· NOAA Digital Coast (http://csc.noaa.gov/digitalcoast/) for Olympic Peninsula

· National Elevation Dataset (for part of the Olympic peninsula)

Oregon

· DOGAMI lidar data (ftp://lidar.engr.oregonstate.edu/)

California

· NOAA Digital Coast (http://csc.noaa.gov/digitalcoast/)

· San Francisco Bay and Sacramento-San Joaquin Delta DEM

(http://baydeltaoffice.water.ca.gov/modeling/deltamodeling/modelingdata/DEM.cfm)

· Suisun Bay and Delta Bathymetry (http://sfbay.wr.usgs.gov/sediment/delta/)
