# Supplementary material for: Insights into estuary habitat loss in the western United States using a new method for mapping maximum extent of tidal wetlands
Source: PLoS One. 2019 Aug 14;14(8):e0218558. doi: 10.1371/journal.pone.0218558 (PMC6693690; doi:10.1371/journal.pone.0218558)
Supplement: S2 File — (PDF) [file pone.0218558.s002.pdf]

S2. Groundtruthing

To ground-truth 50% exceedance level as a reliable elevation for maximum tidal elevation we cross-referenced tidal inundation, high water levels, and elevations of different vegetation types in 15 estuaries on the Pacific Coast. Survey areas at these sites were chosen because they were relatively undisturbed and because of their proximity to local tide gages. Ground-truthing was important to answer two questions: 1) are particular elevations tidally inundated, and 2) what wetland vegetation type occurs at these elevations? We took advantage of vegetation and fish sampling surveys within estuaries for which we could obtain this information at multiple locations. As shown in Table S2.1, observations at most sites were based on surveys in the field. In Morro Bay and the Nooksack delta sites, field surveys were supplemented with aerial photos, and in Willapa and Tomales Bay sites only aerial photos were used. We performed aerial photo interpretation using the history feature of Google Earth to identify areas exhibiting tidal wetting and drying. To identify vegetation types from aerial photos, local vegetation structure was verified from elevation, locations of mudflats, color and texture of vegetation types, and ground photographs in citations (see Data source).

Elevations of sites were determined using GIS-based on the lidar-based digital elevation models (DEMs) used in this study. The 50% exceedance elevation was determined from outputs of the EBEEM model at closest locations along the 50% exceedance contour to the sample point. Mean high high water (MHHW) and mean high water (MHW) were determined from local gages run by NOAA or by local field biologists. Tidal datums for NOAA gages are often in terms of mean lower low water (https://tidesandcurrents.noaa.gov/stations.html?type=Datums), so for these we converted the tidal datum to NAVD88 based on the published elevation offset. Gages for one estuary (Tomales Bay, CA) lacked NAVD88 references, so to calculate the offset we related NAVD88 to mean low water (MLW) and MHHW based on 9 gages in the region to calculate a predicted NAVD88 offset for Tomales Bay gages given their MHHW and MLW values. Both MLW and MHHW were strongly predictive of the NAVD88 offset (R^2 = 0.91. 0.72, respectively), so for each gage in Tomales Bay we used the average predicted NAVD88 offset based on the two regressions.

Data from all observations, used in Fig. 2 of this publication, are noted below in Table S2.2.

Table S.1. Methods for determining tidal inundation and vegetation types at sites in 15 estuaries on the Pacific Coast.

Estuary	Fig. 2 abbrev.	Tidal inundation verified via	Vegetation type verified via	Data source
Nooksack	No	Field sampling	Field sampling & aerial photos	Lummi Tribe and this study
N. Skagit delta	SkN	Field sampling	Field sampling	Skagit River System Cooperative
S. Skagit delta	SkS	Field sampling	Field sampling	Skagit River System Cooperative
Snohomish	Sn	Field sampling	Field sampling	NW Fisheries Science Center
Nisqually	Ni	Field sampling	Field sampling	Billy Frank National Wildlife Refuge
Willapa	Wi	Aerial photos	Aerial photos	This study
Youngs Bay 1	Yo	Field sampling	Field sampling	Institute of Applied Ecology
Youngs Bay 2	Yo	Field sampling	Field sampling	Institute of Applied Ecology
Tillamook	Ti	Field sampling	Field sampling	Institute of Applied Ecology
South Slough	So	Field sampling	Field sampling	South Slough NERR
Coquille	Co	Field sampling	Field sampling	Institute of Applied Ecology
Tomales Bay	To	Aerial photos	Aerial photos	This study, (1)
Elkhorn Slough	El	Field sampling	Field sampling	Elkhorn Slough NERR
Morro Bay	Mo	Field sampling	Field sampling & aerial photos	This study, (2), (3)
Tijuana	Tj	Field sampling	Field sampling	Tijuana NERR

1. Traut BH. The role of coastal ecotones: a case study of the salt marsh/upland transition zone in California. Journal of Ecology. 2005 Apr 1;93(2):279-90.

2. Kitajima, A. & Gillespie, A. Morro Bay National Estuary Program's Data Summary Report 2012. Morro Bay National Estuary Program. October 2012. https://www.mbnep.org/wp-content/uploads/2014/12/2012_Data_Summary_Report.pdf

3. Thorne KM, MacDonald GM, Ambrose RF, Buffington KJ, Freeman CM, Janousek CN, et al. Effects of climate change on tidal marshes along a latitudinal gradient in California: U.S. Geological Survey Open-File Report 2016-1125, 75p.; 2016. doi: http://dx.doi.org/10.3133/ofr20161125.

Table S2.2. Average elevations obtained from tide gages and GIS outputs from lidar DEMs and EBEEM mapping for sites in 15 Pacific Coast estuaries. All elevations are relative to NAVD88.

Estuary	Average vegetated wetland elevation (m)				Local 50% Exceed. (m)	Local MHHW (m)	Local MHW (m)	Tide gage(s)
	Forested	Scrub-shrub	High marsh	Low marsh				
Nooksack	2.97	3.13	2.21	1.64	3.15	2.45	2.23	Bellingham (NOAA)
N. Skagit delta	2.79	3.02	--	2.57	3.07	2.86	2.68	Local field
S. Skagit delta	--	2.58	2.83	--	3.08	2.80	2.61	Local field
Snohomish	2.87	2.48	--	1.98	3.45	2.95	2.78	Local field
Nisqually	3.12	2.99	3.09	2.87	3.70	3.10	2.80	Local field
Willapa	2.71	3.16	3.05	--	3.63	2.56	2.34	South Bend (NOAA)
Youngs Bay 1	--	3.28	2.96	1.73	3.43	2.74	2.53	Local field
Youngs Bay 2	2.83	2.70	2.84	2.12	3.43	2.83	2.61	Local field
Tillamook	2.93	3.08	2.79	2.22	3.32	2.44	2.23	Local field
South Slough	2.62	--	2.51	2.23	2.90	2.21	2.02	Local field
Coquille	2.77	--	2.34	1.88	2.81	2.17	1.95	Local field
Tomales Bay		2.35	2.12	1.91	2.57	1.77	1.54	Average of : Inverness, Sand Point, Reynolds (NOAA)*
Elkhorn Slough	--	--	2.10	1.69	2.15	1.60	1.38	Average of : Elkhorn Slough at Railroad Bridge, Elkhorn (NOAA)
Morro Bay	--	2.33	2.20	1.71	2.22	1.65	1.44	Local field
Tijuana	--	2.11	1.71	1.51	2.13	1.63	1.47	Local field

* Tide gage MHHW and MHW converted from MLLW to NAVD88 datum via regression predictions from 9 tide gages in the region with NAVD88 datum.
