# Supplementary material for: Insights into estuary habitat loss in the western United States using a new method for mapping maximum extent of tidal wetlands
Source: PLoS One. 2019 Aug 14;14(8):e0218558. doi: 10.1371/journal.pone.0218558 (PMC6693690; doi:10.1371/journal.pone.0218558)
Supplement: S3 File — (PDF) [file pone.0218558.s003.pdf]

**S3. Geoprocessing steps for indirect assessment of West Coast USA tidal wetland loss**

We used the following geoprocessing steps in GIS to determine wetland loss within estuaries.

**1. Intersection of Estuary Extent with NWI:** Intersect PMEP's Estuary Extent layer with NWI.

**2. Exclusion of nonvegetated water bodies, mudflats, channels, etc:** Flag polygons that are classified in NWI as nonvegetated or aquatic bed habitats within the Estuarine, Marine, and Riverine systems, and nonvegetated or aquatic bed habitats within the Palustrine and Lacustrine systems with tidal water regime modifiers. These aren't considered in the loss assessment (except as described in Step 5 below), because they were probably nonvegetated historically, and this analysis includes only vegetated tidal wetlands (emergent, shrub and forested classes). However, the wetlands flagged in this step may be classified as "lost" under certain conditions (see step 5 below).

**3. Identification of areas that are classified as tidal wetlands in NWI and are therefore considered "retained":** In the intersected layer, look at the non-flagged areas from step 2 (in NWI, these are either classified as vegetated = EM, SS, or FO; or upland). Within these areas, consider all areas classified in NWI as tidal wetlands or tidally-influenced wetlands to be "retained." These are areas classified in the Estuarine system in NWI, or in other systems with tidally-influenced hydrology ("tidal water regime modifier"). However, even within these areas, wetlands with special modifiers indicating diking, drainage or farming are considered "lost" (Table 3).

**4. Areas considered "lost" by default:** All areas not considered "retained" are considered "lost." These include areas mapped as nontidal wetlands or uplands in NWI.

**5. Inclusion of areas that are currently nonvegetated, but which were probably vegetated tidal wetlands prior to major alterations:** Add to the "lost" category all nonvegetated Estuarine wetlands with NWI modifiers indicating diked, drained, or farmed status ("h", "d", and "f"), and all nonvegetated Palustrine and Lacustrine wetlands that lack a tidal water regime modifier. This step produces more accurate results for former tidal marshes in the San Francisco Bay area converted to salt ponds (which were originally tidal marshes), and other impounded former tidal marshes and swamps that are now lakes or ponds. Most of these wetlands are now unvegetated due to land use practices and/or subsidence.

**6. Upper boundary correction:** Flag polygons that are not mapped in the NWI (i.e. areas considered upland in NWI) and which have the following 3 characteristics: a) long, narrow polygons; b) adjacent to a tidal water body or tidal wetland; and c) adjacent to the upslope boundary of the Estuary Extent. These are classified as "retained;" they are a product of the differences in scale and methods used in our analysis vs. NWI. These polygons, which form a sort of "bathtub ring" around NWI tidal wetland features, are now recognized as tidal wetlands in PMEP's Estuary Extent mapping due to their elevation. However, they are not easily detectable using the NWI protocol, which relies heavily on aerial photo analysis and other existing data sources, rather than detailed elevation analysis. This analysis added over 34,000 polygons totaling around 14,000 acres – an important correction that helps minimize errors due to scale and methods discrepancies.
