# Supplementary material for: Insights into estuary habitat loss in the western United States using a new method for mapping maximum extent of tidal wetlands
Source: PLoS One. 2019 Aug 14;14(8):e0218558. doi: 10.1371/journal.pone.0218558 (PMC6693690; doi:10.1371/journal.pone.0218558)
Supplement: S4 File — (PDF) [file pone.0218558.s004.pdf]

S4. Estuaries defined and used in analysis

Summary and locations of 444 Pacific Coast estuaries classified by CMECS biogeographic setting (Salish Sea, Washington, Oregon, and Northern California Coast, Central California, and Southern California Bight ecoregions) and physiographic setting (embayment, major river delta, riverine estuary, and lagoonal estuary). Historical extent of vegetated tidal wetlands (emergent, shrub and tidal classes) and their losses are included for the 55 estuaries analyzed, as well as the year of the most recent update to the National Wetland Inventory (NWI) estimate of current vegetated wetland extent. Sites are mapped by ecoregion using the Estuary ID on Figures S4.1 through S4.4. All data in this publication are available online at https://psmfc.sharefile.com/d-s5bf1b1efca24e7eb. As the dataset is updated, the revised geodatabase can be found at https://pacificfishhabitat.org.

Estuary ID	Estuary Name	CMECS Physiographic Setting	Historical Estuary Extent (ha)	Historical Extent - Vegetated (ha)	Current Extent - Vegetated (ha)	Vegetated Loss %	NWI Year
**Salish Sea (Figure S4.1)**							
1000	Point Roberts	Embayment/Bay	85.2				1980
1001	Drayton Harbor	Embayment/Bay	969.9				1981
1002	Birch Bay	Embayment/Bay	677.3	146.4	19.2	86.9	1981
1003	Nooksack River	Major River Delta	6,421.2	2,729.1	274.2	90.0	1981
1004	Padden Creek	Embayment/Bay	3.1				1981
1005	Chuckanut Bay	Embayment/Bay	45.2				1981
1006	Nelson Bay	Embayment/Bay	64.2				1980
1007	Westcott Bay	Embayment/Bay	79.5				1980
1008	Rocky Bay	Embayment/Bay	4.6				1981
1009	Garrison Bay	Embayment/Bay	65.9				1980
1010	Blind Bay	Embayment/Bay	52.0				1980
1011	Squaw Bay	Embayment/Bay	22.9				1980
1012	Argyle Lagoon	Lagoonal Estuary	6.1				1981
1013	Fisherman Bay	Embayment/Bay	214.5				1980
1014	False Bay	Embayment/Bay	105.6				1981
1015	Davis Bay	Embayment/Bay	45.8				1980
1016	Barlow Bay	Embayment/Bay	62.9				1980
1017	Samish Bay	Major River Delta	6,685.5	3,336.0	59.8	98.2	1981
1018	Padilla Bay	Embayment/Bay	8,553.9	2,691.1	111.7	95.9	1981
1019	Fidalgo Bay	Embayment/Bay	910.0				1981
1020	Ship Harbor Lagoon	Lagoonal Estuary	5.3				1981
1021	Flounder Bay	Embayment/Bay	19.8				1981
1022	Simik Bay	Embayment/Bay	337.7				1981
1023	Bowman Bay	Embayment/Bay	38.9				1981

1024	Dugualla Bay	Embayment/Bay	653.7	245.5	11.4	95.4	1981
1025	Skagit Bay	Major River Delta	17,144.6	9,803.0	1,705.3	82.6	1981
1026	Crescent Harbor Salt Marsh	Lagoonal Estuary	105.3				1981
1027	Freund Marsh	Lagoonal Estuary	30.5				1981
1028	Maylor Point	Lagoonal Estuary	25.8				1981
1029	Grasser's Lagoon	Lagoonal Estuary	7.8				1981
1030	Kennedys Lagoon	Lagoonal Estuary	5.1				1981
1031	Perigo's Lagoon	Lagoonal Estuary	18.6				1981
1032	Harrington Lagoon	Lagoonal Estuary	4.1				1981
1033	Race Lagoon	Lagoonal Estuary	15.5				1981
1034	Stillaguamish River	Major River Delta	9,954.3	3,124.1	911.2	70.8	1981
1035	Triangle Cove	Lagoonal Estuary	111.5				1980
1036	Crockett Lake	Lagoonal Estuary	367.8				1981
1037	Camano Country Club	Lagoonal Estuary	9.3				1980
1038	Saratoga Pass Tidelands	Lagoonal Estuary	8.2				1981
1039	Elger Bay	Lagoonal Estuary	31.6				1980
1040	Lake Hancock	Lagoonal Estuary	81.8				1981
1041	Lagoon Point	Embayment/Bay	23.0				1981
1042	Tulalip Bay	Embayment/Bay	156.6				1980
1043	Snohomish River	Major River Delta	9,438.1	6,330.9	672.9	89.4	1981
1044	Deer Lagoon / Useless Bay	Embayment/Bay	664.8	371.0	14.8	96.0	1981
1045	Maxwelton	Lagoonal Estuary	84.3				1980
1046	Cultus Bay	Embayment/Bay	358.9				1980
1047	Appletree Cove	Embayment/Bay	58.1				1981
1048	Miller Bay	Riverine Estuary	183.5				1980
1049	Doe-Kag-Wats	Lagoonal Estuary	16.0				1981
1050	Liberty Bay	Embayment/Bay	385.0				1981
1051	Point Monroe	Lagoonal Estuary	18.1				1981
1052	Keyport Lagoon	Embayment/Bay	27.7				1981
1053	Port Madison	Embayment/Bay	70.8				1981
1054	Salmon Bay	Riverine Estuary	18.4				2000
1055	Burke Bay	Riverine Estuary	20.8				1981
1056	Fletcher Bay	Embayment/Bay	22.4				1981
1057	Clear Creek	Embayment/Bay	69.3				1981
1058	Barker Creek	Riverine Estuary	26.8				1981
1059	Eagle Harbor	Embayment/Bay	106.8				1981
1060	Chico Creek	Riverine Estuary	58.9				1981
1061	Schel-chelb	Embayment/Bay	30.2				1981
1062	Phinney Bay	Embayment/Bay	41.8				1981
1063	Oyster Bay	Embayment/Bay	79.6				1981
1064	Clam Bay	Embayment/Bay	48.6				1981
1065	Duwamish River	Major River Delta	491.1				2000
1066	Gorst Creek	Embayment/Bay	81.4				1981
1067	Curley Creek	Riverine Estuary	48.5				1981

1068	Harper	Embayment/Bay	21.7				1981
1069	Miller Creek	Riverine Estuary	39.2				1980
1070	Olalla Creek	Riverine Estuary	25.8				1981
1071	Gig Harbor	Embayment/Bay	52.0				1981
1072	Puyallup River	Major River Delta	544.6				1980
1073	Burley Lagoon	Embayment/Bay	232.3				1981
1074	Minter Creek	Riverine Estuary	75.7				1981
1075	Glen Cove	Embayment/Bay	35.3				1981
1076	Wollochet Bay	Embayment/Bay	46.1				1981
1077	Days Island Harbor	Embayment/Bay	18.1				1981
1078	Chambers Creek	Riverine Estuary	40.6				1981
1079	West Filucy Bay	Embayment/Bay	8.6				1981
1080	East Oro Bay	Embayment/Bay	50.5				1981
1081	Oro Bay	Embayment/Bay	55.9				1981
1082	Nisqually River	Major River Delta	1,968.6	979.2	298.9	69.5	1981
1083	Mill Bight	Embayment/Bay	14.1				1980
1084	Taylor Bay	Embayment/Bay	18.1				1980
1085	Henderson Inlet	Embayment/Bay	331.4				1980
1086	Big Fishtrap	Embayment/Bay	19.2				1980
1087	Gull Harbor	Embayment/Bay	25.4				1980
1088	Deschutes River	Major River Delta	380.8				1980
1089	Mud Bay	Embayment/Bay	399.1				1980
1090	Young Cove	Embayment/Bay	29.1				1980
1091	Fry Cove	Embayment/Bay	14.1				1980
1092	Sanderson Harbor	Embayment/Bay	12.9				1980
1093	Hudson Cove	Embayment/Bay	65.2				1981
1094	Oyster Bay	Embayment/Bay	687.4				1981
1095	Skookum Inlet	Embayment/Bay	287.5				1981
1096	Belspeox Point	Lagoonal Estuary	4.3				1980
1097	Mill Creek	Riverine Estuary	13.1				1980
1098	Goldsborough Creek	Riverine Estuary	86.1				1981
1099	Campbell Creek / Chapman Cove	Embayment/Bay	73.0				1981
1100	Oakland Bay	Embayment/Bay	279.8				1981
1101	West Harstine Island	Embayment/Bay	10.9				1980
1102	Whiteman Cove	Lagoonal Estuary	14.0				1980
1103	Herron	Embayment/Bay	15.2				1980
1104	Jarrell Cove	Embayment/Bay	24.2				1980
1105	Dutcher Cove	Embayment/Bay	36.5				1980
1106	McLane Cove	Embayment/Bay	24.5				1980
1107	Grapeview	Embayment/Bay	14.0				1980
1108	Vaughn Bay	Embayment/Bay	93.3				1980
1109	Rocky Bay	Embayment/Bay	116.7				1980
1110	North Bay	Embayment/Bay	399.5				1980
1111	Lynch Cove	Embayment/Bay	678.0	170.9	129.4	24.3	1980

1112	Tahuya River	Riverine Estuary	105.0				1980
1113	Skokomish River	Major River Delta	1,035.2	394.8	202.0	48.8	1980
1114	Dewatto Bay	Riverine Estuary	58.1				1980
1115	Lillwaup Bay	Riverine Estuary	32.2				1980
1116	Hamma Hamma River	Major River Delta	186.3				1982
1117	Anderson Creek	Riverine Estuary	31.9				1980
1118	Duckabush River	Major River Delta	205.0				1980
1119	Stavis Bay	Embayment/Bay	64.8				1980
1120	Seabeck Bay	Embayment/Bay	40.2				1980
1121	Big Beef Creek	Riverine Estuary	65.7				1980
1122	Pleasant Harbor	Embayment/Bay	16.1				1980
1123	Anderson Creek	Riverine Estuary	47.3				1980
1124	Fisherman Harbor	Embayment/Bay	22.9				1980
1125	Dosewallips River	Major River Delta	350.0				1980
1126	Zelatched Point Lagoon	Lagoonal Estuary	6.8				1980
1127	Right Smart Cove	Embayment/Bay	17.3				1980
1128	Jackson Cove	Embayment/Bay	32.5				1980
1129	Bangor Lake	Lagoonal Estuary	4.2				1981
1130	Cattail Lake	Lagoonal Estuary	5.0				1981
1131	Quilcene Bay	Major River Delta	446.8	126.0	106.1	15.8	1980
1132	Broad Spit	Lagoonal Estuary	4.9				1980
1133	Thorndyke Creek	Riverine Estuary	110.8				1981
1134	Tarboo Bay	Embayment/Bay	221.5				1980
1135	Port Gamble	Embayment/Bay	182.6				1981
1136	Bridgehaven	Lagoonal Estuary	25.7				1981
1137	Shine Creek	Riverine Estuary	46.1				1981
1138	Coon Bay	Embayment/Bay	27.7				1981
1139	Foulweather Bluff	Lagoonal Estuary	7.8				1981
1140	Twin Spits	Lagoonal Estuary	14.5				1981
1141	Port Ludlow	Embayment/Bay	28.3				1981
1142	Mats Mats Bay	Embayment/Bay	57.9				1981
1143	Kilisut Harbor	Embayment/Bay	22.4				1981
1144	Oak Bay	Embayment/Bay	95.2				1981
1145	Hadlock	Embayment/Bay	27.4				1981
1146	Chimacum Creek	Riverine Estuary	35.9				1980
1147	Walan Point	Lagoonal Estuary	9.5				1981
1148	Port Townsend mill	Lagoonal Estuary	6.9				1980
1149	Salmon-Snow	Riverine Estuary	114.4				1980
1150	Gardiner	Lagoonal Estuary	8.0				1980
1151	Sequim Bay	Embayment/Bay	1,403.1				1980
1152	Gierin Creek	Riverine Estuary	479.6				1980
1153	Dungeness Bay	Major River Delta	1,524.3				1980
1154	McDonald Creek	Riverine Estuary	12.0				1980
1155	Morse Creek	Riverine Estuary	12.5				1981

1156	The Lagoon	Lagoonal Estuary	11.4				1981
1157	Elwha River	Major River Delta	133.9				1981
1158	Salt Creek	Riverine Estuary	56.2				1981
1159	East Twin River	Riverine Estuary	5.7				1981
1160	Pysht River	Riverine Estuary	266.1				1981
1161	Clallam River	Riverine Estuary	19.1				1981
1162	Hoko River	Riverine Estuary	58.6				1981
1163	Sekiu River	Riverine Estuary	49.2				1981
1164	Bullman Creek	Riverine Estuary	24.4				1981
1165	Sail River	Riverine Estuary	4.0				1981
**WA, OR, Northern CA Coast (Figure S4.2)**							
2000	Waatch River	Riverine Estuary	162.4	122.0	120.8	1.0	1981
2001	Sooes River	Riverine Estuary	71.6				1981
2002	Ozette River	Riverine Estuary	3.6				1981
2003	Quillayute River	Riverine Estuary	188.4				1981
2004	Goodman Creek	Riverine Estuary	8.7				1981
2005	Mosquito Creek	Riverine Estuary	2.0				1981
2006	Hoh River	Riverine Estuary	182.5				1981
2007	Cedar Creek	Riverine Estuary	2.1				1981
2008	Kalaloch Creek	Riverine Estuary	5.9				1981
2009	Queets River	Riverine Estuary	203.7				1981
2010	Whale Creek	Riverine Estuary	2.1				1981
2011	Raft River	Riverine Estuary	43.1				1981
2012	Camp Creek	Riverine Estuary	2.8				1981
2013	Duck Creek	Riverine Estuary	1.4				1981
2014	Quinault River	Riverine Estuary	173.5				1981
2015	Wreck Creek	Riverine Estuary	3.5				1981
2016	Moclips River	Riverine Estuary	23.3				1981
2017	Joe Creek	Riverine Estuary	24.2				1981
2018	Elk Creek	Riverine Estuary	5.4				1981
2019	Boone Creek	Riverine Estuary	3.6				1981
2020	Copalis River	Riverine Estuary	219.7				1981
2021	Connor Creek	Riverine Estuary	54.4				1981
2022	Grays Harbor	Riverine Estuary	33,583.2	8,711.9	4,753.8	45.4	1981
2023	Willapa Bay	Riverine Estuary	43,264.3	8,040.0	4,780.6	40.5	2011
2024	Loomis Lake Creek	Riverine Estuary	104.7				2011
2025	Columbia River - Reach A	Riverine Estuary	20,903.9	6,291.0	1,063.0	83.1	2011
2026	Columbia River - Reach B	Riverine Estuary	34,094.7	9,252.3	4,782.1	48.3	1981
2027	Columbia River - Reach C	Riverine Estuary	17,294.4	10,403.0	1,695.7	83.7	1981
2028	Columbia River - Reach D	Riverine Estuary	4,354.3	2,338.8	156.2	93.3	1981
2029	Columbia River - Reach E	Riverine Estuary	5,951.9	3,413.1	211.0	93.8	1981
2030	Columbia River - Reach F	Riverine Estuary	21,127.6	12,184.8	3,686.9	69.7	2009
2031	Columbia River - Reach G	Riverine Estuary	8,976.0	3,319.2	544.6	83.6	1981
2032	Columbia River - Reach H	Riverine Estuary	3,639.1	541.9	259.6	52.1	2011

2033	Clatsop Spit	Riverine Estuary	20.6				2000
2034	Necanicum River	Riverine Estuary	273.0				2000
2035	Ecola Creek	Riverine Estuary	22.0				2000
2036	Nehalem River	Riverine Estuary	2,126.0	1,196.4	546.5	54.3	2000
2037	Lake Lytle	Lagoonal Estuary	33.7				2000
2038	Smith Lake	Lagoonal Estuary	1.1				2000
2039	Tillamook Bay	Riverine Estuary	5,677.1	2,292.1	494.2	78.4	2000
2040	Netarts Bay	Embayment/Bay	1,065.9	126.0	119.8	4.9	2000
2041	Sand Lake	Lagoonal Estuary	476.4				2000
2042	Nestucca Bay	Riverine Estuary	1,119.4	660.3	134.7	79.6	2014
2043	Daley Lake	Riverine Estuary	8.4				2014
2044	Neskowin Creek	Riverine Estuary	4.3				2014
2045	Salmon River	Riverine Estuary	356.8	254.6	229.9	9.7	2014
2046	Devils Lake	Riverine Estuary	0.3				2010
2047	Siletz Bay	Riverine Estuary	1,097.2	467.1	242.8	48.0	2010
2048	Depoe Bay	Riverine Estuary	4.7				2010
2049	Little Creek	Riverine Estuary	4.8				2010
2050	Yaquina Bay	Riverine Estuary	2,690.9	983.2	295.3	70.0	2010
2051	Beaver Creek	Riverine Estuary	97.2				2010
2052	Alsea Bay	Riverine Estuary	1,441.6	496.0	367.0	26.0	2010
2053	Big Creek	Riverine Estuary	10.1				2000
2054	Yachats River	Riverine Estuary	25.6				2000
2055	Tenmile Creek	Riverine Estuary	1.7				2000
2056	Big Creek	Riverine Estuary	1.5				2000
2057	Cape Creek	Riverine Estuary	0.5				2000
2058	Berry Creek	Riverine Estuary	0.3				2000
2059	Sutton Creek	Riverine Estuary	12.1				2000
2060	Siuslaw River	Riverine Estuary	2,557.7	1,445.0	648.0	55.2	2000
2061	Siltcoos River	Riverine Estuary	32.1				2000
2062	Tahkenitch Creek	Riverine Estuary	32.8				2000
2063	Umpqua River	Riverine Estuary	5,025.6	1,942.9	861.9	55.6	2000
2064	Tenmile Creek	Riverine Estuary	537.8				2000
2065	Coos Bay	Riverine Estuary	8,322.8	3,286.5	896.0	72.7	2000
2066	Sunset Bay	Embayment/Bay	0.9				2000
2067	Coquille River	Riverine Estuary	4,378.5	3,497.0	157.1	95.5	2011
2068	Twomile Creek	Riverine Estuary	24.6				2011
2069	Fourmile Creek	Riverine Estuary	33.2				2011
2070	New River	Lagoonal Estuary	51.9				2000
2071	Floras Creek	Riverine Estuary	9.7				2000
2072	Sixes River	Lagoonal Estuary	43.3				2000
2073	Elk River	Lagoonal Estuary	62.0				2000
2074	Port Orford Head	Riverine Estuary	57.1				2000
2075	Hubbard Creek	Riverine Estuary	0.8				2000
2076	Brush Creek	Riverine Estuary	2.0				2000

2077	Mussel Creek	Riverine Estuary	1.0				2000
2078	Euchre Creek	Riverine Estuary	13.2				2000
2079	Rogue River	Riverine Estuary	357.9				2000
2080	Hunter Creek	Riverine Estuary	13.1				2000
2081	Myers Creek	Riverine Estuary	1.4				2000
2082	Pistol River	Lagoonal Estuary	40.0				2000
2083	Thomas Creek	Riverine Estuary	0.9				2000
2084	Whaleshead Creek	Riverine Estuary	2.6				2000
2085	Chetco River	Riverine Estuary	97.7				2000
2086	Winchuck River	Lagoonal Estuary	19.8				2000
2087	Smith River	Riverine Estuary	481.3	130.7	29.8	77.2	1983
2088	Lake Earl	Lagoonal Estuary	1,609.4				1983
2089	Pebble Beach	Lagoonal Estuary	14.5				1983
2090	Crescent City Harbor	Embayment/Bay	189.0				1983
2091	NC9 Cresent Beach	Lagoonal Estuary	7.1				1983
2092	Wilson Creek	Lagoonal Estuary	0.7				2010
2093	Lagoon Creek	Lagoonal Estuary	17.5				2010
2094	Klamath River	Riverine Estuary	531.8				2010
2095	Johnson Creek	Lagoonal Estuary	1.5				2010
2096	Ossagon Creek	Lagoonal Estuary	66.8				2010
2097	Fern Canyon	Lagoonal Estuary	30.8				2010
2098	Squashan Creek	Lagoonal Estuary	1.6				2010
2099	Espa Lagoon	Lagoonal Estuary	4.8				2010
2100	Redwood Creek	Lagoonal Estuary	64.9				2010
2101	Stone Lagoon	Lagoonal Estuary	264.1				2010
2102	Big Lagoon	Lagoonal Estuary	723.3				2010
2103	Little River	Lagoonal Estuary	45.0				2010
2104	Clam Beach	Lagoonal Estuary	2.9				2010
2105	Mad River	Lagoonal Estuary	195.5				2010
2106	Humboldt Bay	Embayment/Bay	10,683.3	3,777.1	532.3	85.9	2010
2107	Eel River	Riverine Estuary	4,275.5	2,991.2	447.8	85.0	2010
2108	Guthrie Creek	Lagoonal Estuary	1.3				2010
2109	Bear River	Lagoonal Estuary	18.4				2002
**Central California (Figure S4.3)**							
3000	McNutt Gulch	Lagoonal Estuary	1.0				2002
3001	Mattole River	Lagoonal Estuary	167.6				2002
3002	Jackass Creek	Lagoonal Estuary	1.9				2002
3003	Usal Creek	Lagoonal Estuary	7.5				2002
3004	Cottaneva Creek	Lagoonal Estuary	3.2				2002
3005	Wages Creek	Lagoonal Estuary	3.2				2002
3006	Chadbourne Gulch	Lagoonal Estuary	0.6				2002
3007	Seaside Creek	Lagoonal Estuary	5.4				2002
3008	Ten Mile River	Lagoonal Estuary	87.7				2002
3009	Inglenook Fen	Lagoonal Estuary	12.2				2002

3010	Lake Cleone	Lagoonal Estuary	9.0				2002
3011	Virgin Creek	Lagoonal Estuary	5.6				2002
3012	Pudding Creek	Lagoonal Estuary	28.7				2002
3013	Noyo River	Riverine Estuary	41.5				2002
3014	Hare Creek	Lagoonal Estuary	4.1				2002
3015	Mitchell Creek	Lagoonal Estuary	1.8				2002
3016	Caspar Creek	Lagoonal Estuary	4.5				2002
3017	Russian Gulch	Lagoonal Estuary	5.5				2002
3018	Big River	Riverine Estuary	128.7				2002
3019	Little River	Lagoonal Estuary	6.6				2002
3020	Albion River	Riverine Estuary	76.5				2002
3021	Little Salmon Creek	Lagoonal Estuary	1.6				2002
3022	Navarro River	Lagoonal Estuary	75.7				2002
3023	Greenwood Creek	Lagoonal Estuary	5.6				2002
3024	Elk Creek	Lagoonal Estuary	16.7				2002
3025	Irish Gulch	Riverine Estuary	0.7				2002
3026	Alder Creek	Lagoonal Estuary	6.3				2002
3027	Manchester	Lagoonal Estuary	27.7				2002
3028	Brush Creek	Lagoonal Estuary	41.6				2002
3029	Garcia River	Riverine Estuary	43.7				2002
3030	Gualala River	Lagoonal Estuary	51.0				2002
3031	Stump Beach	Lagoonal Estuary	1.0				2002
3032	Russian Gulch Creek	Lagoonal Estuary	4.0				2002
3033	Russian River	Lagoonal Estuary	369.1				2002
3034	Salmon Creek	Lagoonal Estuary	32.7				1985
3035	Bodega Bay	Embayment/Bay	391.5				1985
3036	Estero Americano	Lagoonal Estuary	217.1				1985
3037	Estero de San Antonio	Lagoonal Estuary	86.4				1985
3038	Tomales Bay	Embayment/Bay	3,384.7				1985
3039	Horseshoe Pond	Lagoonal Estuary	17.4				1985
3040	Drakes Estero	Embayment/Bay	1,118.3	282.0	274.5	2.7	1985
3041	Bolinas Lagoon	Embayment/Bay	510.4	136.2	117.0	14.1	1985
3042	Redwood Creek/Big Lagoon	Lagoonal Estuary	4.1				2009
3043	Tennessee Valley Lagoon	Lagoonal Estuary	1.8				2009
3044	Rodeo Lagoon	Lagoonal Estuary	20.9				2009
3045	Sacramento-San Joaquin Delta	Major River Delta	174,212.0	154,033.0	4,964.3	96.8	2012
3046	Suisun-Grizzly Bays	Embayment/Bay	37,832.8	25,539.7	3,262.5	87.2	2009
3047	San Pablo Bay	Embayment/Bay	57,510.4	25,016.2	6,185.0	75.3	2009
3048	San Francisco Bay	Embayment/Bay	24,164.1	1,207.2	385.4	68.1	2009
3049	South San Francisco Bay	Embayment/Bay	74,972.1	25,182.4	3,480.9	86.2	2009
3050	Calera Creek	Riverine Estuary	1.0				2009
3051	San Pedro Creek	Lagoonal Estuary	0.6				1985
3052	Half Moon Bay Airport Lagoon	Lagoonal Estuary	3.6				2009

3053	Pillar Point Harbor	Embayment/Bay	146.6				2009
3054	Frenchmans Creek	Lagoonal Estuary	2.9				1985
3055	Pilarcitos Creek	Lagoonal Estuary	7.4				1985
3056	Tunitas Creek	Lagoonal Estuary	3.2				1993
3057	San Gregorio Creek	Lagoonal Estuary	9.4				1993
3058	Pomponio Creek	Lagoonal Estuary	1.7				1993
3059	Pescadero Creek	Lagoonal Estuary	129.1				1993
3060	Lake Lucerne	Lagoonal Estuary	17.8				1993
3061	Gazos Creek	Lagoonal Estuary	1.3				1993
3062	Waddell Creek	Lagoonal Estuary	16.9				1993
3063	Scott Creek Lagoon	Lagoonal Estuary	12.4				1993
3064	Laguna Creek	Lagoonal Estuary	5.8				1993
3065	Baldwin Creek	Lagoonal Estuary	7.2				1993
3066	Lombardi Creek	Lagoonal Estuary	0.8				1993
3067	Dairy Gulch	Lagoonal Estuary	0.9				1993
3068	Wilder Creek	Lagoonal Estuary	11.5				1993
3069	Younger Lagoon	Lagoonal Estuary	2.6				1993
3070	San Lorenzo River	Lagoonal Estuary	16.7				1993
3071	Santa Cruz Harbor	Embayment/Bay	27.3				1993
3072	Schwan Lagoon	Lagoonal Estuary	11.7				1981
3073	Corcoran Lagoon	Lagoonal Estuary	8.7				1981
3074	Moran Lake	Lagoonal Estuary	1.4				1981
3075	Soquel Creek	Lagoonal Estuary	3.0				1981
3076	Aptos Creek	Lagoonal Estuary	1.6				1981
3077	Pajaro River	Lagoonal Estuary	367.7				2005
3078	Elkhorn Slough	Embayment/Bay	2,398.0	1,662.6	511.7	69.2	2005
3079	Salinas River	Lagoonal Estuary	211.0				2005
3080	Carmel River	Lagoonal Estuary	37.6				2005
3081	Garrapata Creek	Lagoonal Estuary	0.6				2005
3082	Little Sur Lagoon	Lagoonal Estuary	15.2				2005
3083	Big Sur River	Riverine Estuary	7.3				2005
3084	San Carpoforo Creek	Lagoonal Estuary	8.9				2005
3085	Arroyo de la Cruz	Lagoonal Estuary	9.2				2005
3086	Arroyo del Corral	Lagoonal Estuary	1.7				2005
3087	Arroyo Laguna	Lagoonal Estuary	1.1				2005
3088	Arroyo del Puerto	Lagoonal Estuary	0.6				2005
3089	Little Pico Creek	Lagoonal Estuary	1.3				2005
3090	Pico Creek	Lagoonal Estuary	2.9				2005
3091	San Simeon Creek	Lagoonal Estuary	6.2				2005
3092	Santa Rosa Creek	Lagoonal Estuary	6.4				2005
3093	Villa Creek Lagoon	Lagoonal Estuary	3.2				2005
3094	Cayucos Creek Lagoon	Lagoonal Estuary	1.5				2005
3095	Old Creek	Lagoonal Estuary	1.0				2005
3096	Alva Paul Creek	Lagoonal Estuary	1.3				2005

3097	Morro Creek Lagoon	Lagoonal Estuary	2.5				2005
3098	Morro Bay	Embayment/Bay	1,046.4	211.3	207.2	2.0	2005
3099	San Luis Obispo Creek Lagoon	Lagoonal Estuary	17.2				2005
3100	Pismo Creek Lagoon	Lagoonal Estuary	12.4				2005
3101	Arroyo Grande Creek Lagoon	Lagoonal Estuary	17.7				2012
3102	Santa Maria River	Lagoonal Estuary	126.4				2012
3103	San Antonio Creek	Lagoonal Estuary	10.4				2005
3104	Santa Ynez River	Lagoonal Estuary	238.2				2005
3105	Honda Creek Lagoon	Lagoonal Estuary	1.3				2005
3106	Jalama Creek	Lagoonal Estuary	1.0				2006
**Southern California Bight (Figure S4.4)**							
4000	Canada de la Gaviota Creek	Lagoonal Estuary	2.9				2006
4001	Bell Canyon Creek	Lagoonal Estuary	1.1				2006
4002	Devereux Lagoon	Lagoonal Estuary	25.8				2006
4003	Goleta Slough	Lagoonal Estuary	144.4				2006
4004	Arroyo Burro	Lagoonal Estuary	0.6				2006
4005	Mission Creek Lagoon	Lagoonal Estuary	3.5				2006
4006	Sycamore Creek	Lagoonal Estuary	0.7				2006
4007	Andree Clark Bird Refuge	Lagoonal Estuary	13.3				2006
4008	Carpinteria Salt Marsh	Embayment/Bay	88.5				2006
4009	Carpinteria Creek	Lagoonal Estuary	0.9				2006
4010	Ventura River	Lagoonal Estuary	17.2				2002
4011	Ventura Marina	Embayment/Bay	77.2				2006
4012	Santa Clara River	Lagoonal Estuary	130.1				2006
4013	Channel Islands Harbor	Embayment/Bay	146.5				2006
4014	Port Hueneme	Embayment/Bay	59.6				2006
4015	Ormond Beach	Lagoonal Estuary	30.3				2006
4016	Mugu Lagoon	Riverine Estuary	1,321.7	980.2	491.1	49.9	2006
4017	Trancas	Lagoonal Estuary	0.8				2006
4018	Zuma Canyon	Lagoonal Estuary	1.8				2006
4019	Malibu Lagoon	Lagoonal Estuary	13.7				2006
4020	Santa Monica Canyon	Riverine Estuary	0.7				2006
4021	Marina del Rey	Embayment/Bay	255.7				2006
4022	Ballona Creek	Embayment/Bay	137.5				2006
4023	Cabrillo Marina	Embayment/Bay	109.0				2006
4024	Long Beach Harbor	Embayment/Bay	1,486.2				2005
4025	Los Angeles Harbor	Embayment/Bay	1,660.1				2006
4026	Los Angeles River	Riverine Estuary	166.8				2005
4027	Long Beach Marina	Embayment/Bay	38.3				2005
4028	East San Pedro Bay	Embayment/Bay	2,600.5				2005
4029	Alamitos Bay	Embayment/Bay	343.1	144.4	19.2	86.7	2005
4030	San Gabriel River	Riverine Estuary	153.2				2005
4031	Anaheim Bay	Embayment/Bay	763.0	418.6	265.9	36.5	2005

4032	Bolsa Bay	Embayment/Bay	209.9				2005
4033	Bolsa Chica Lowlands	Embayment/Bay	875.9				2005
4034	Huntington Channel	Embayment/Bay	625.4	582.3	27.6	95.3	2006
4035	Santa Ana River	Riverine Estuary	130.5				2006
4036	Newport Bay	Embayment/Bay	808.8	279.4	162.3	41.9	2006
4037	Aliso Creek	Lagoonal Estuary	1.6				2006
4038	San Juan Creek	Lagoonal Estuary	10.5				2002
4039	Dana Point Harbor	Embayment/Bay	69.1				2002
4040	San Mateo Lagoon	Lagoonal Estuary	4.3				2002
4041	San Onofre Creek	Lagoonal Estuary	2.0				2002
4042	Las Pulgas Creek	Lagoonal Estuary	5.8				2002
4043	Las Flores Creek	Lagoonal Estuary	3.5				2002
4044	Aliso Canyon	Lagoonal Estuary	6.6				2002
4045	French Canyon	Lagoonal Estuary	8.8				2002
4046	Cockleburr Canyon	Lagoonal Estuary	0.7				2002
4047	Santa Margarita Lagoon	Lagoonal Estuary	184.2				2002
4048	Oceanside Harbor	Embayment/Bay	92.2				2002
4049	San Luis Rey River	Lagoonal Estuary	18.8				2002
4050	Loma Alta Marsh	Lagoonal Estuary	1.6				2002
4051	Buena Vista Lagoon	Lagoonal Estuary	94.3				2002
4052	Agua Hedionda	Embayment/Bay	153.0				2002
4053	Batiquitos Lagoon	Embayment/Bay	217.6				2002
4054	San Elijo Lagoon	Lagoonal Estuary	158.2				2002
4055	San Dieguito Lagoon	Embayment/Bay	137.5				2002
4056	Los Penasquitos Lagoon	Lagoonal Estuary	95.5				2002
4057	Mission Bay	Embayment/Bay	924.8				2002
4058	San Diego River	Riverine Estuary	147.4				2002
4059	San Diego Bay	Embayment/Bay	5,195.9	679.2	166.2	75.5	2005
4060	Tijuana River	Riverine Estuary	332.4	263.3	249.8	5.1	2005

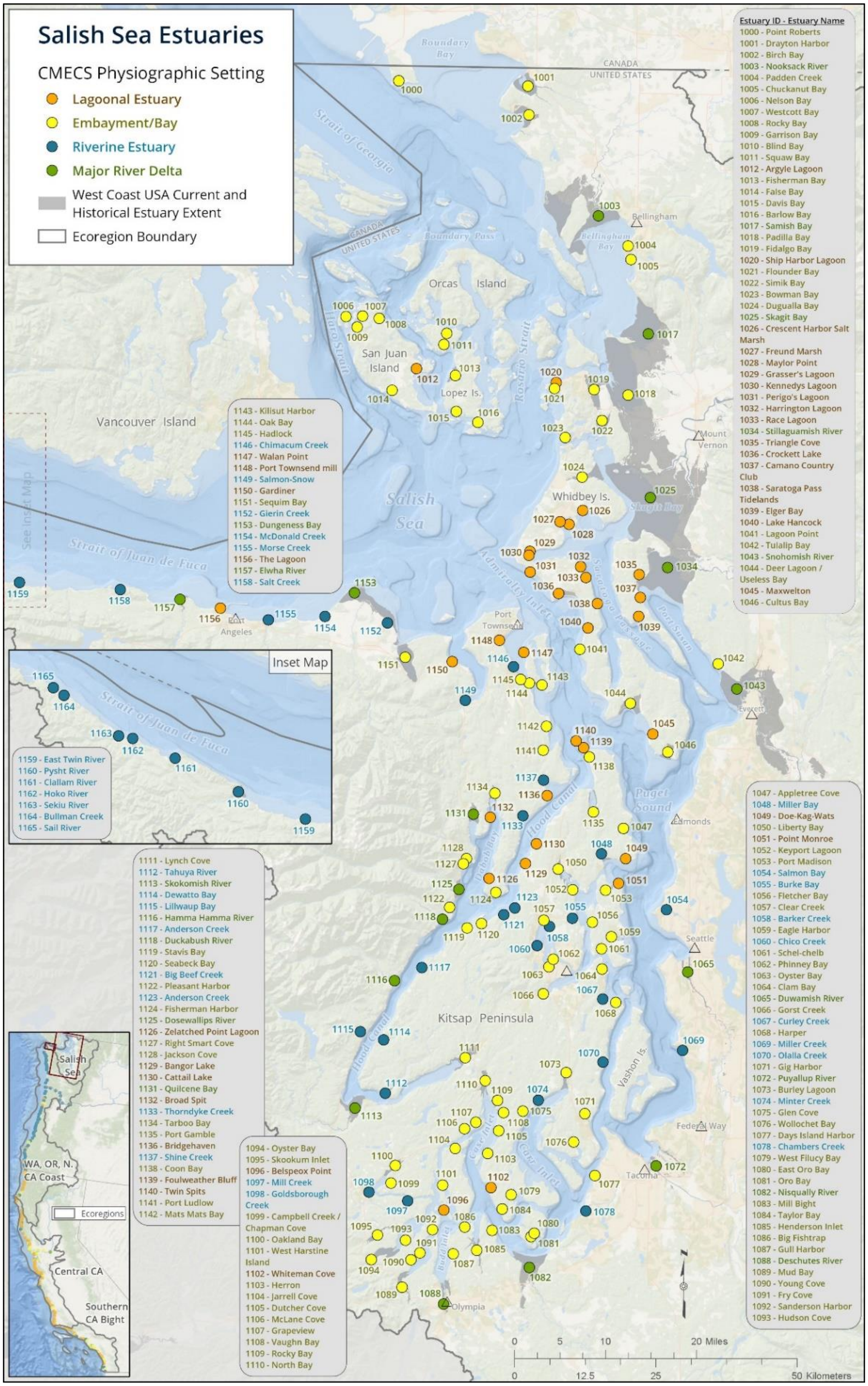

Figure S4.1. Estuaries of the Salish Sea.

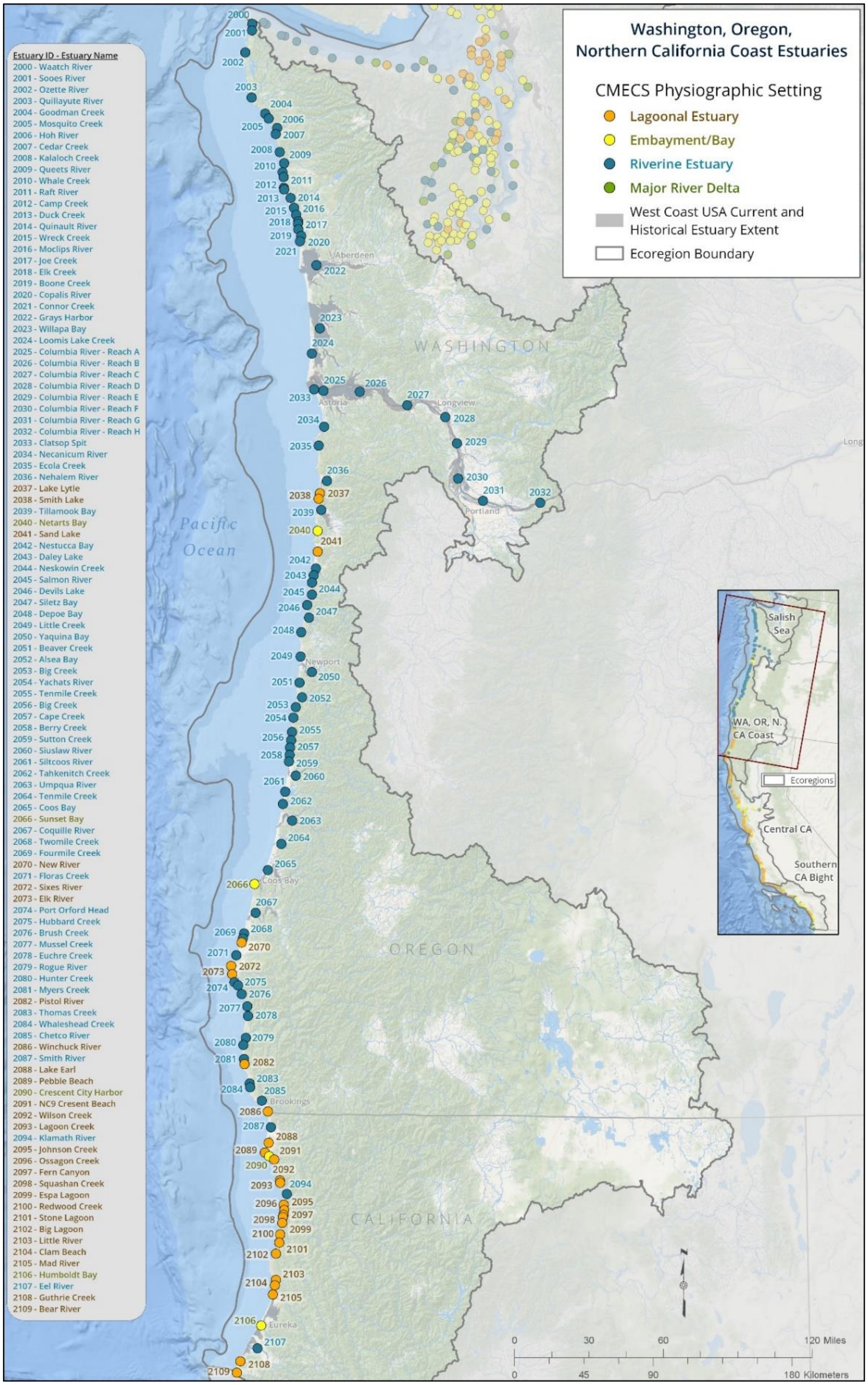

Figure S4.2. Estuaries of the Washington-Oregon-Northern California Coast.

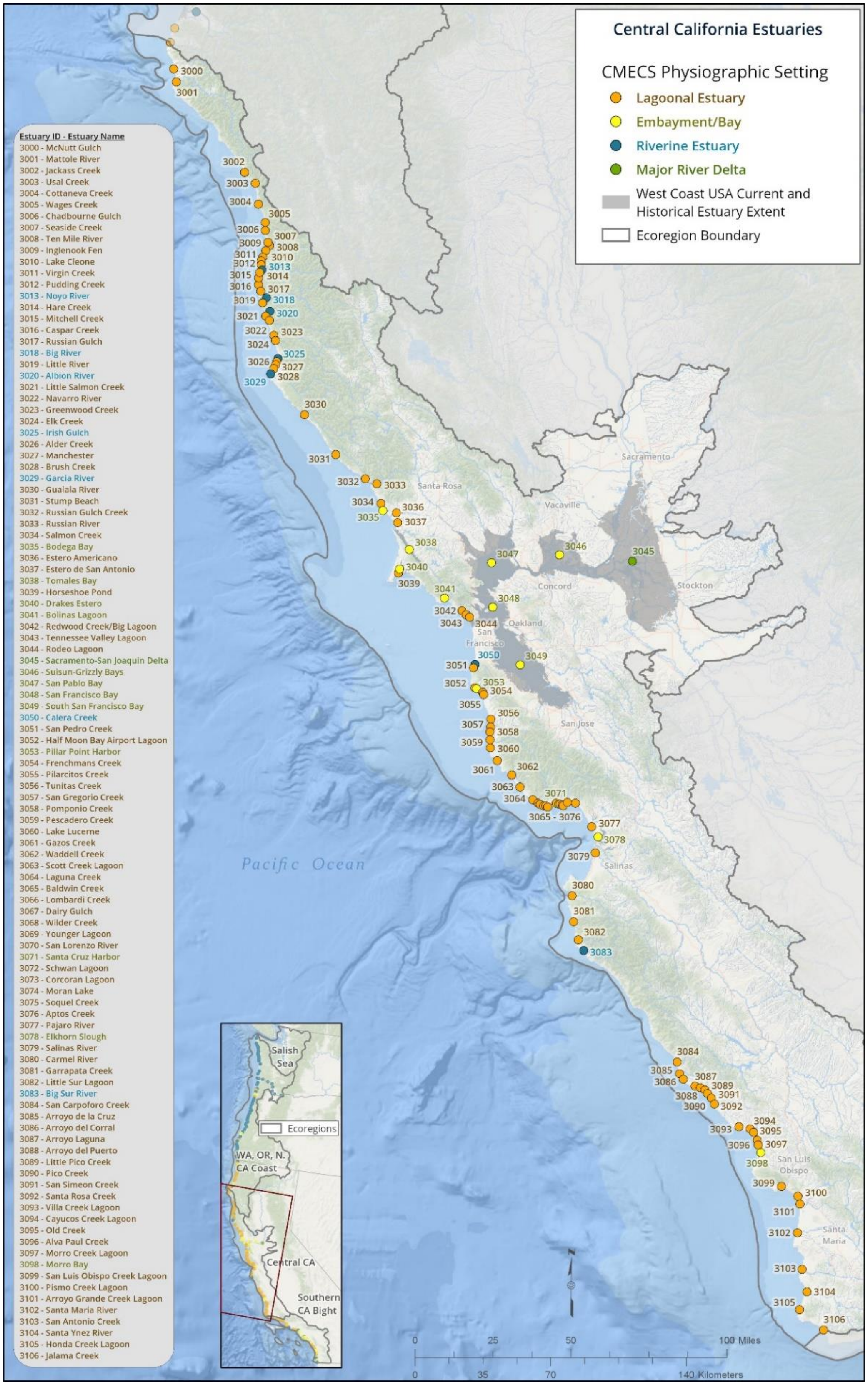

Figure S4.3. Estuaries of Central California.

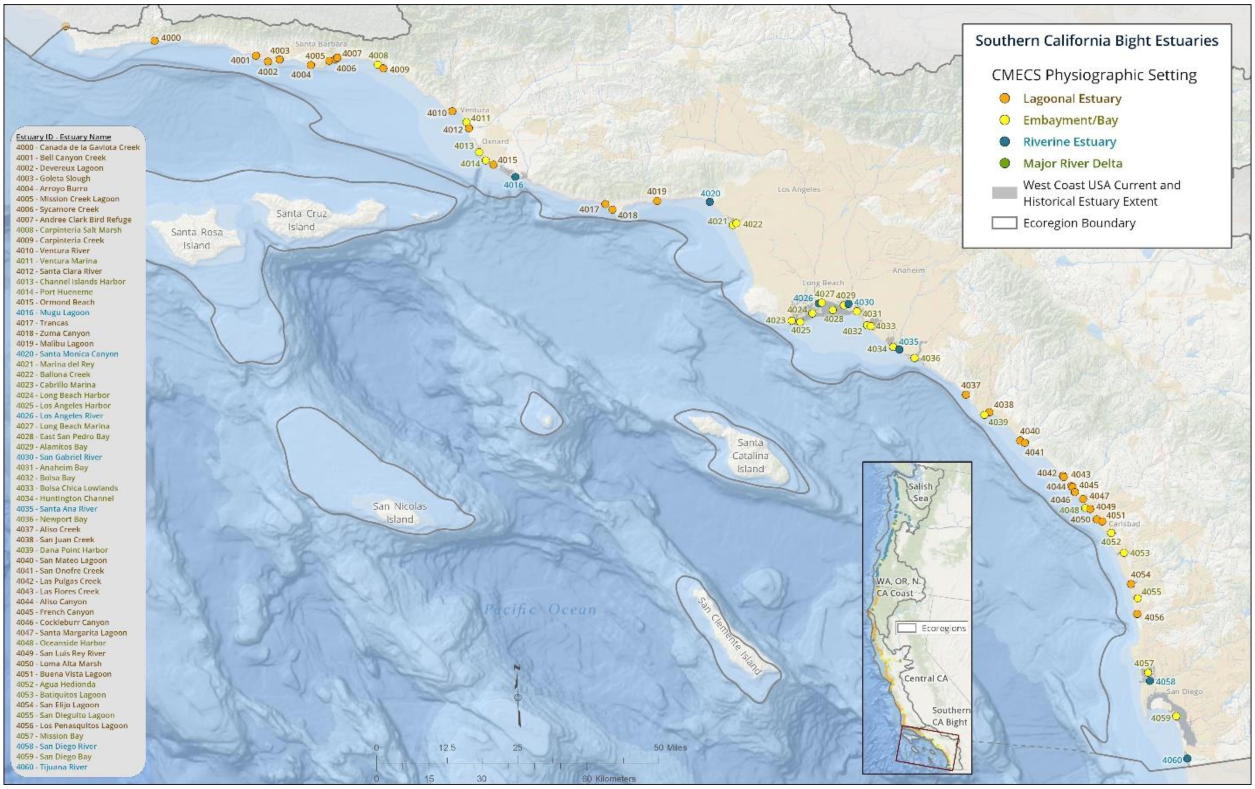

Figure S4.4. Estuaries of the Southern California Bight.
